# *Pseudomonas aeruginosa* surface motility and invasion into competing communities enhance interspecies antagonism

**DOI:** 10.1128/mbio.00956-24

**Published:** 2024-08-06

**Authors:** Andrea Sánchez-Peña, James B. Winans, Carey D. Nadell, Dominique H. Limoli

**Affiliations:** 1 Department of Microbiology and Immunology, Carver College of Medicine, University of Iowa, Iowa City, Iowa, USA; 2 Department of Biological Sciences, Dartmouth College, Hanover, New Hampshire, USA; 3 Department of Biology, Indiana University, Bloomington, Indiana, USA; University of Washington, Seattle, Washington, USA

**Keywords:** *Pseudomonas aeruginosa*, *Staphylococcus aureus*, type IV pili motility, spatial organization, polymicrobial, biofilms

## Abstract

Chronic polymicrobial infections involving *Pseudomonas aeruginosa* and *Staphylococcus aureus* are prevalent, difficult to eradicate, and associated with poor health outcomes. Therefore, understanding interactions between these pathogens is important to inform improved treatment development. We previously demonstrated that *P. aeruginosa* is attracted to *S. aureus* using type IV pili (TFP)-mediated chemotaxis, but the impact of attraction on *S. aureus* growth and physiology remained unknown. Using live single-cell confocal imaging to visualize microcolony structure, spatial organization, and survival of *S. aureus* during coculture, we found that interspecies chemotaxis provides *P. aeruginosa* a competitive advantage by promoting invasion into and disruption of *S. aureus* microcolonies. This behavior renders *S. aureus* susceptible to *P. aeruginosa* antimicrobials. Conversely, in the absence of TFP motility, *P. aeruginosa* cells exhibit reduced invasion of *S. aureus* colonies. Instead, *P. aeruginosa* builds a cellular barrier adjacent to *S. aureus* and secretes diffusible, bacteriostatic antimicrobials like 2-heptyl-4-hydroxyquinoline-*N*-oxide (HQNO) into the *S. aureus* colonies. Reduced invasion leads to the formation of denser and thicker *S. aureus* colonies with increased HQNO-mediated lactic acid fermentation, a physiological change that could complicate treatment strategies. Finally, we show that *P. aeruginosa* motility modifications of spatial structure enhance competition against *S. aureus*. Overall, these studies expand our understanding of how *P. aeruginosa* TFP-mediated interspecies chemotaxis facilitates polymicrobial interactions, highlighting the importance of spatial positioning in mixed-species communities.

**IMPORTANCE:**

The polymicrobial nature of many chronic infections makes their eradication challenging. Particularly, coisolation of *Pseudomonas aeruginosa* and *Staphylococcus aureus* from airways of people with cystic fibrosis and chronic wound infections is common and associated with severe clinical outcomes. The complex interplay between these pathogens is not fully understood, highlighting the need for continued research to improve management of chronic infections. Our study unveils that *P. aeruginosa* is attracted to *S. aureus*, invades into neighboring colonies, and secretes anti-staphylococcal factors into the interior of the colony. Upon inhibition of *P. aeruginosa* motility and thus invasion, *S. aureus* colony architecture changes dramatically, whereby *S. aureus* is protected from *P. aeruginosa* antagonism and responds through physiological alterations that may further hamper treatment. These studies reinforce accumulating evidence that spatial structuring can dictate community resilience and reveal that motility and chemotaxis are critical drivers of interspecies competition.

## INTRODUCTION

Microorganisms exist in complex polymicrobial environments, such as the soil and the human body, where they interact with each other and respond to changes in their surroundings (1–3). These interactions can lead to the emergence of community-level properties not observed in monoculture (4–10). The resulting collective behavior can have significant implications for the microbial physiology, evolution, and interactions with the host. For example, bacterial pathogens can enhance both virulence and antibiotic tolerance in mixed communities (2, 4, 11–16), potentially undermining current chronic infection treatments.

*Pseudomonas aeruginosa* and *Staphylococcus aureus* are the most prevalent and abundant pathogens in individuals with cystic fibrosis (CF) (5, 17) and persist in significant quantities in the lungs for decades (18). Critically, their coinfection is linked with more severe lung disease, increased rates of hospitalization, and reduced lung function in patients (19–23). Additionally, coinfection in chronic burn wounds can delay healing time (24). Thus, there is a need to further understand how interactions between these two organisms exacerbate the outcomes of polymicrobial infections.

Several *in vitro* studies support clinical observations that *P. aeruginosa* and *S. aureus* increase each other’s virulence during coculture (12, 25). *P. aeruginosa* secretes numerous anti-staphylococcal factors including the siderophores pyoverdine and pyochelin, phenazines, rhamnolipids, staphylolytic proteases like LasA, and quinolones (26–32). Many of these secreted products alter *S. aureus* physiology, enhancing its antibiotic tolerance (13, 15, 16, 33–35). One example is the small molecule, 2-heptyl-4-hydroxyquinoline *N*-oxide (HQNO), which inhibits *S. aureus* cellular respiration, shifting its metabolism to fermentation (11, 30, 36, 37). HQNO has been detected in CF sputum (30) and can increase *S. aureus* tolerance to several antibiotics used clinically (13, 15, 16). However, the effect of these antimicrobials (AM) on *S. aureus* has been mainly studied in the presence of *P. aeruginosa* cell-free spent medium in a well-mixed environment (13, 15, 16, 35, 38). Interestingly, recent studies found that HQNO modifies the spatial organization of *P. aeruginosa* and *S. aureus* in a synthetic CF sputum medium (SCFM2) (39) and chronic murine wounds (40), highlighting the importance of visualizing communities in a structured environment. Therefore, to elucidate how interspecies interactions negatively impact clinical outcomes, experimental models are needed that better reflect how microbes naturally encounter each other, namely, under spatial constraint.

Previously, we demonstrated that *P. aeruginosa* responds to *S. aureus* from a distance by increasing type IV pili (TFP) motility, mediated by retractile appendages that allow *P. aeruginosa* to move across surfaces through twitching motility (41). Specifically, when *P. aeruginosa* and *S. aureus* start as spatially separated single cells, we observed that *P. aeruginosa* uses the Pil-Chp chemoreceptor, PilJ, to respond from a distance by directionally moving toward *S. aureus* (42, 43). This attraction requires secretion of *S. aureus* peptides called phenol-soluble modulins (42, 44). However, it remains unknown how *P. aeruginosa* chemotaxis toward *S. aureus* influences *S. aureus* physiology and survival.

Here, we sought to understand the consequences of *P. aeruginosa* TFP-mediated attraction on *S. aureus*. We visualized *P. aeruginosa* and *S. aureus* interactions at the single-cell level over time using resonant scanning confocal microscopy and discovered that *P. aeruginosa* utilizes a combination of TFP-mediated motility and secreted antimicrobials to effectively outcompete *S. aureus* under these conditions. Particularly, we found that wild-type (WT) *P. aeruginosa* is attracted to, invades, and disrupts *S. aureus* colonies. Moreover, *P. aeruginosa*-secreted antimicrobials HQNO, pyoverdine, pyochelin, and LasA were necessary for negatively influencing *S. aureus* growth, but not for invasion and disruption of *S. aureus* colonies. Conversely, in the absence of TFP motility, *P. aeruginosa* cannot invade *S. aureus* colonies but rather grows around them, leading to an altered *S. aureus* colony architecture resulting in compact, thicker colonies with increased biomass compared with coculture with WT *P. aeruginosa*. In addition to these effects on *S. aureus* colony architecture, we also found that coculture with a TFP-deficient *P. aeruginosa* leads to altered physiology through the induction of an HQNO-mediated increase in *S. aureus* fermentation. Moreover, TFP motility was crucial for modulating the spatial arrangement and competitive dynamics between *P. aeruginosa* and *S. aureus* in conditions that capture essential features of the CF airway environment. Overall, our findings highlight the importance of spatial organization in community-based behaviors and the need for a more thorough understanding of the interplay between polymicrobial communities in the context of infection.

## RESULTS

### TFP are necessary for *P. aeruginosa* invasion into *S. aureus* colonies

**Fig 1:**
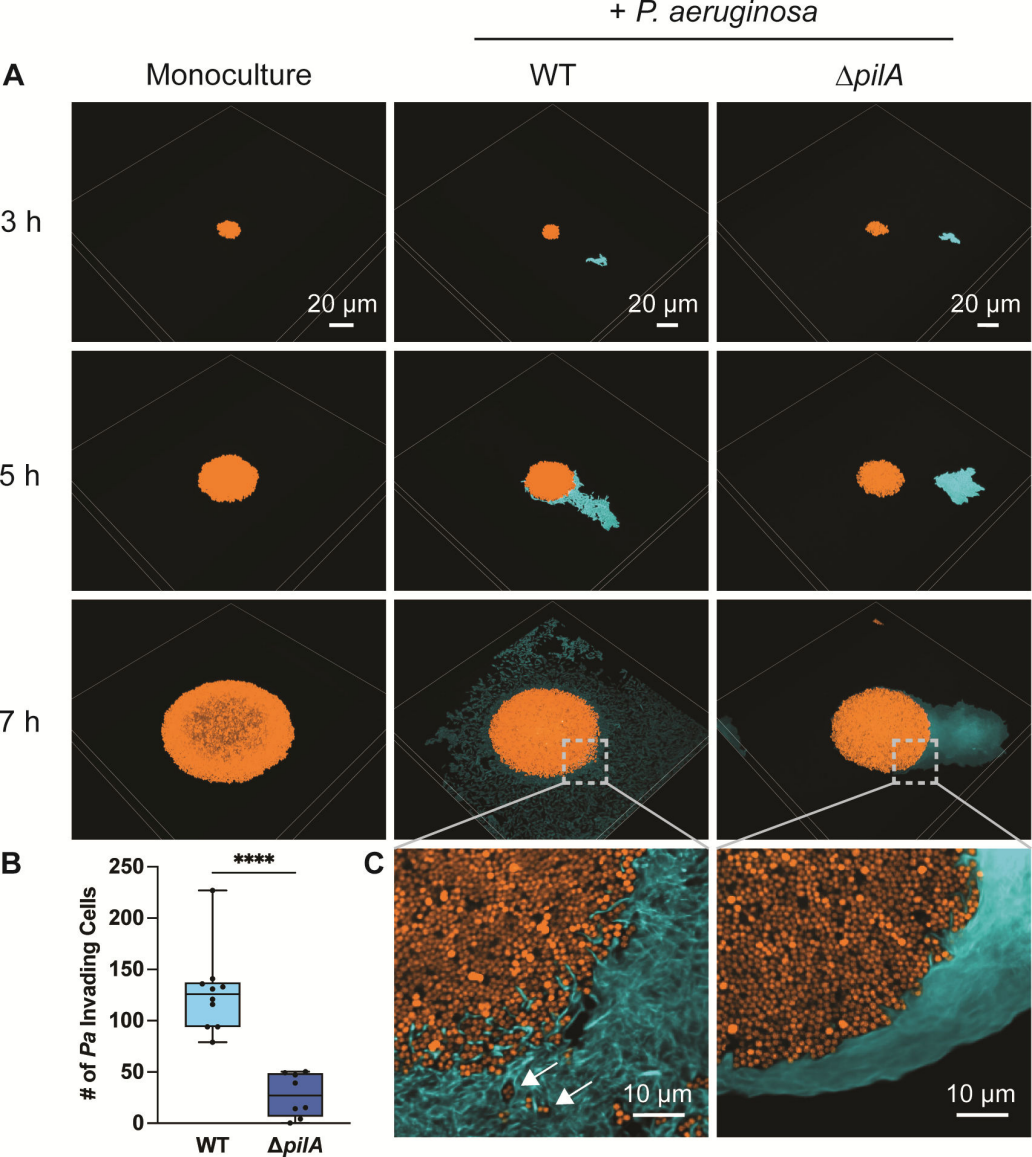
Type IV pili are necessary for *P. aeruginosa* invasion into *S. aureus* colonies. Resonant scanning confocal live imaging of *S. aureus* and *P. aeruginosa*. (A) Representative micrographs of WT *S. aureus* (pseudocolored orange) in monoculture or in coculture with *P. aeruginosa* (pseudocolored cyan; WT or TFP-deficient mutant Δ*pilA*). (B) Quantification of *P. aeruginosa* single-cell invasion into *S. aureus* colonies *t* ~ 7 hours in mono- or coculture with *P. aeruginosa* (WT or Δ*pilA*). At least four biological replicates with two technical replicates each were analyzed. Each data point represents one technical replicate. Statistical significance was determined by a Mann-Whitney *U*-test. ****P < 0.0001. (C) Zoomed micrograph of *S. aureus* colony edge in coculture with *P. aeruginosa* (WT or Δ*pilA*). White arrows indicate dispersed *S. aureus* cells. *S. aureus*: pCM29 P*_sarA_*_P1-*sgfp*_; *P. aeruginosa*: chromosomal P*_A1/04/03-mCherry_*.

We previously reported that *P. aeruginosa* responds to *S. aureus* from a distance by using TFP to chemotax toward and surround *S. aureus* colonies (42), but how this behavior affects *S. aureus* physiology remained unclear. To test the consequences of *P. aeruginosa* chemotaxis on *S. aureus*, we first visualized *P. aeruginosa* interactions with *S. aureus* colonies in three dimensions by performing live resonant scanning confocal microscopy of *S. aureus* in mono- or coculture with *P. aeruginosa* WT or a TFP-deficient mutant (∆*pilA*). Here, *S. aureus* and *P. aeruginosa* constitutively expressed *sgfp* (pseudocolored orange) and *mCherry* (pseudocolored cyan), respectively. Bacteria were inoculated between a cover slip and an agarose pad for visualization in the same visual field over time. Imaging was initiated with *S. aureus* and *P. aeruginosa* as single cells, positioned approximately 30 to 50 µm apart to provide sufficient time and distance for *P. aeruginosa* to respond to the presence of *S. aureus*. As previously demonstrated (42), at approximately 5 hours, we observed that WT *P. aeruginosa* responds to *S. aureus* by breaking into single cells and moving toward it with TFP motility, which eventually leads to *P. aeruginosa* surrounding, invading, and disrupting *S. aureus* cells from the colony (Fig. 1). This invasion is dependent on TFP motility, as *P. aeruginosa* ∆*pilA* exhibited significantly decreased invasion compared with WT (Fig. 1B). While the ∆*pilA* mutant is amotile, it eventually grows against the *S. aureus* colony at later time points (Fig. 1A and C). These data suggest that TFP motility is not only necessary for *P. aeruginosa* chemotaxis toward *S. aureus* but also enables effective invasion of *P. aeruginosa* into *S. aureus* colonies.

### *P. aeruginosa* TFP motility-mediated invasion influences *S. aureus* growth and architecture

To investigate how *P. aeruginosa* invasion affects *S. aureus* colony physiology, we imaged *S. aureus* in mono- or coculture with WT or ∆*pilA P. aeruginosa* following ~24 hours of incubation. At later time points, visualizing *P. aeruginosa* becomes challenging due to reduced fluorescence from decreased *mCherry* production and photobleaching. Nevertheless, phase contrast microscopy confirmed that *P. aeruginosa* cells surround *S. aureus* colonies after ~24 hours (Fig. S1).

**Fig 2:**
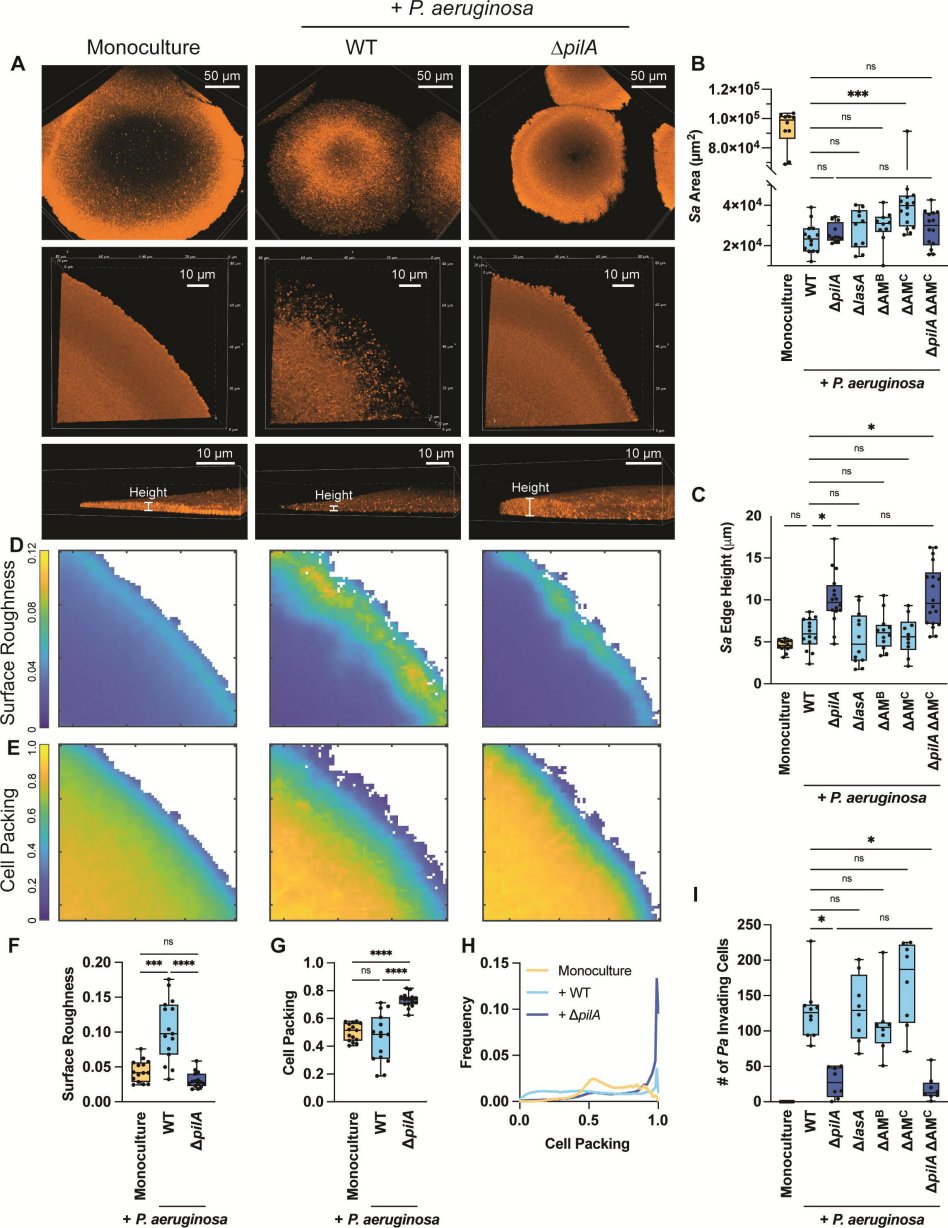
*P. aeruginosa* type IV pili motility-mediated invasion influences the architecture of *S. aureus* colonies independently of *P. aeruginosa*-secreted antimicrobials. Analysis of *S. aureus* colony edge disruption and thickness. (A) Representative resonant scanning confocal micrographs of the whole colony (top row) or Galvano scanner colony edge micrographs of WT *S. aureus* (orange) in monoculture or coculture with *P. aeruginosa* (not shown; WT or Δ*pilA*) at *t* ~ 24 hours shown from the top (middle row) of the colony or the side (bottom row). The micrographs in A (bottom row) show the colonies on the Z-plane and demonstrate how the height was quantified. Quantification of *S. aureus* whole colony area at *t* ~ 24 hours (B) or height at the edge of *S. aureus* (*Sa*) colony (µm) at *t* ~ 24 hours (C) in monoculture or coculture with *P. aeruginosa* (WT, Δ*pilA*, Δ*lasA*, ΔAM^B^ [bacteriostatic antimicrobials; HQNO, pyoverdine, and pyochelin], ΔAM^C^ [complete antimicrobials; HQNO, pyoverdine, pyochelin, and LasA], or Δ*pilA* ΔAM^C^). (D–G) Representative BiofilmQ heatmaps (D and E) and quantification (F and G) of local surface roughness and cell packing analysis at *S. aureus* colony edge in mono- or coculture with *P. aeruginosa* (WT or Δ*pilA*). Each data point represents the average of two technical replicates within one biological replicate. Statistical significance was determined by a Mann-Whitney *U*-test with an *ad hoc* Bonferroni correction for multiple comparisons. (H) Cell packing distribution within *S. aureus* colony edge in the abovementioned conditions. A Kolmogorov-Smirnov cumulative distribution test was performed, and all three conditions were significantly different (****P < 0.0001) from one another. A total of 15 biological replicates with two technical replicates each were analyzed in panels F–H. (I) The number of invading *P. aeruginosa* (*Pa*) single cells inside *S. aureus* was quantified at *t* ~ 7 hours in mono- or coculture with the *P. aeruginosa* strains described above. At least four biological replicates with two technical replicates each were analyzed per condition in panels B, C, and I. Each data point represents one technical replicate. Statistical significance in panels B, C, and I was determined by Kruskal-Wallis followed by Dunn’s multiple comparisons test. n.s., not significant; *P < 0.05, ***P < 0.001, and ****P < 0.0001.

We found that in coculture with WT *P. aeruginosa*, *S. aureus* forms smaller colonies than in monoculture by measuring the area at the base of the *S. aureus* colony (Fig. 2A and B). Moreover, *P. aeruginosa* TFP motility-mediated invasion resulted in *S. aureus* colony edges exhibiting reduced fluorescence, likely caused by dispersed, lysed cells, or a combination thereof (Fig. 2A). In the presence of ∆*pilA*, the area of *S. aureus* colonies was comparable to that in the presence of WT *P. aeruginosa* (Fig. 2B). However, despite similar growth area, *S. aureus* colonies exhibited less dispersal at the colony edges in coculture with ∆*pilA*, possibly due to loss of *P. aeruginosa* invasion (Fig. 2A; top and middle rows). Additionally, *S. aureus* colonies appeared thicker and denser than in coculture with WT *P. aeruginosa*, likely due to reduced cell dispersal.

To further investigate this, we visualized and quantified *S. aureus* colony architecture in more detail. Since thickness and density were more distinct on the colony edges, images were acquired with higher magnification and spatial resolution using galvanometric point-scanning confocal microscopy at the end time point (Fig. 2A; middle row). We then measured the height at the edge of *S. aureus* colonies at 15 µm from the edge using the Z-plane (Fig. 2A, bottom row, and Fig. 2C). As expected, the height at the colony edge was significantly higher in coculture with ∆*pilA* than in mono- or coculture with WT *P. aeruginosa* (Fig. 2C). To quantitatively analyze colony density, we measured both cell packing and colony surface roughness using the microscopy image analysis software BiofilmQ (45). These parameters quantify density by measuring the amount of surface or volume within a specified area. In BiofilmQ, *S. aureus* colony edges were separated from the background by segmentation onto a 3D grid, with each cubic grid unit measuring 0.72 µm per side. Neighborhood surface roughness and cell packing were then calculated by determining the biovolume fraction and surface height variance of *S. aureus* for each grid cube within 4 and 6 µm, respectively. Representative heatmaps in Fig. 2D and E provide a two-dimensional visualization of the quantified data in Fig. 2F through H, using color coding to represent local surface roughness and cell packing. *S. aureus* colonies in monoculture show low surface roughness and uniform cell packing (Fig. 2D and E [first column] and Fig. 2F and G). Conversely, when WT *P. aeruginosa* is present, *S. aureus* edges exhibit significantly increased surface roughness and slightly decreased cell packing (Fig. 2D and E [middle column] and Fig. 2F and G), which suggests reduced colony density is caused by WT *P. aeruginosa*. When cocultured with ∆*pilA* (i.e., lacking invasion and colony disruption), *S. aureus* colonies portrayed significantly reduced colony surface roughness and increased cell packing compared with WT *P. aeruginosa* coculture (Fig. 2D and E [last column] and Fig. 2F and G). While the colony cell roughness was not different between *S. aureus* coculture with ∆*pilA* and monoculture, the mean colony cell packing was significantly increased (Fig. 2F and G). Additionally, we analyzed the cell packing distribution within *S. aureus* colony edges and found all three conditions to be statistically different (Fig. 2H). The majority of the monoculture colony edge population was distributed between 0.5 and 1.0, with almost no low-density areas. In *S. aureus* coculture with ∆*pilA*, the large peak at 1.0 indicates that the majority of cells within these edges are highly packed. On the other hand, in the presence of WT *P. aeruginosa*, the cell packing is more evenly distributed with an increased proportion of the population at low-density values compared with monoculture or coculture with ∆*pilA*. However, there is also an increased proportion of the population at high-density values portrayed as a small peak at 1.0. This peak may be attributed to the colony edge height being slightly higher than monoculture *S. aureus* as reported in Fig. 2C, leading to higher cell packing as this calculation considers the three-dimensional space.

Thus, while the base area of the colony is similar in the presence of WT or ∆*pilA*, the colonies are more densely packed when *P. aeruginosa* lacks TFP. Altogether, these observations reveal the crucial role of *P. aeruginosa* TFP motility in altering *S. aureus* architecture. Without TFP motility, *P. aeruginosa* does not invade or disrupt *S. aureus* colonies; instead, it grows alongside them, resulting in increased compaction and altered *S. aureus* colony structure.

### *P. aeruginosa* secretes antimicrobials that affect *S. aureus* growth but do not influence *S. aureus* colony architecture

Next, we wondered how invasion changes *S. aureus* colony architecture and enhances competition. One hypothesis is that invasion increases the local concentration of *P. aeruginosa* antimicrobials within *S. aureus* colonies. Additionally, these anti-staphylococcal factors could aid *P. aeruginosa* invasion. If the former is correct, *S. aureus* colonies grown in the presence of the *P. aeruginosa* ∆*pilA* would be protected from *P. aeruginosa* antimicrobials. *P. aeruginosa* secretes many factors known to inhibit or lyse *S. aureus*, including HQNO, a respiratory toxin that inhibits the *S. aureus* electron transport chain (30, 36), the siderophores pyoverdine and pyochelin, which aid in iron scavenging (29, 32), and an anti-staphylococcal protease, staphylolysin or LasA, which lyses *S. aureus* by cleaving the peptidoglycan pentaglycine cross-links (31).

Since ∆*pilA* had reduced invasion and disruption of *S. aureus* exterior structure compared with WT, we first determined if this difference is due to variations in levels of secreted antimicrobials between the *P. aeruginosa* strains and tested whether ∆*pilA* produces similar levels of exoproducts as WT. The cell-free supernatant from ∆*pilA* or WT *P. aeruginosa* was added to *S. aureus* to examine its growth and lysis over time. No differences were observed in either *S. aureus* lysis or growth rate when exposed to supernatant from WT or ∆*pilA P. aeruginosa*, confirming that each produces similar levels of antimicrobials (Fig. S2). Here, supernatant from *P. aeruginosa* ∆*lasA* served as a control to confirm that staphylolysin is the main driver of *S. aureus* lysis.

To test the hypothesis that *P. aeruginosa* invasion enhances competition by increasing diffusion and local antimicrobial concentration within *S. aureus* colonies, we next examined *S. aureus* colony growth dynamics in the presence of *P. aeruginosa* strains lacking genes encoding antimicrobials. These include a staphylolysin mutant (∆*lasA*), a strain without both HQNO and siderophores (∆*pqsL* ∆*pvdA* ∆*pchE*), referred to as “∆AM^B^” (antimicrobials^Bacteriostatic^), and a mutant with all four antimicrobials deleted (∆*pqsL* ∆*pvdA* ∆*pchE* ∆*lasA*) which we call “∆AM^C^” (AM^Complete^) in Fig. 2B, C and I. The interactions between *S. aureus* and these antimicrobial-deficient strains were assessed by live imaging as described for Fig. 1, and *P. aeruginosa* invasion and *S. aureus* colony height (as a proxy for biomass) were quantified. No detectable differences were observed between coculture with WT *P. aeruginosa* and the antimicrobial mutants for either the *S. aureus* colony edge height (Fig. 2C) or the number of invading cells (Fig. 2I), which suggests that these antimicrobials do not play a role in *P. aeruginosa* invasion or increased *S. aureus* colony height observed in coculture with ∆*pilA*. Yet, it is known that these antimicrobials can impact *S. aureus* growth *in vitro* (11, 13, 15, 16, 35, 37, 38, 46). Therefore, we measured *S. aureus* colony base area to examine antimicrobial influence on growth under these conditions. The *S. aureus* colony area did not significantly increase when cocultured with ∆AM^B^ or ∆*lasA*, compared with the WT (Fig. 2B). However, colony area did increase upon deletion of all four antimicrobials (∆AM^C^), which suggests that while these antimicrobials do not influence *S. aureus* colony architecture, their combinatorial effect alters *S. aureus* growth and colony area.

To determine if *S. aureus* colony edge height and *P. aeruginosa* invasion are driven by motility alone or a combined effect of motility and antimicrobials, we deleted *pilA* in the ∆AM^C^ mutant, generating ∆*pqsL* ∆*pvdA* ∆*pchE* ∆*lasA* ∆*pilA* (∆*pilA* ∆AM^C^). *S. aureus* colony height and invasion of *P. aeruginosa* ∆*pilA* ∆AM^C^ phenocopied ∆*pilA* (Fig. 2C and I), suggesting that TFP motility plays a prominent role in driving these phenotypes. Furthermore, when comparing the effect of ∆*pilA* on the WT background to the ∆AM^C^ background, significant antimicrobial influence on the colony area is only observed when TFP are functional, supporting that motility may enhance antimicrobial action against *S. aureus* under these conditions (Fig. 2B).

Overall, these data suggest that thicker and denser *S. aureus* colony architecture is exclusively mediated by the absence of *P. aeruginosa* TFP-mediated colony invasion and that the main *P. aeruginosa* anti-staphylococcal factors do not substantially influence this observation. Furthermore, *P. aeruginosa* TFP motility may enhance antimicrobial access into the colony to fully affect *S. aureus* growth, revealing the important role *P. aeruginosa* motility plays in antagonistic interactions against *S. aureus*.

### Increased cell packing enhances HQNO-mediated *S. aureus* fermentation

**Fig 3:**
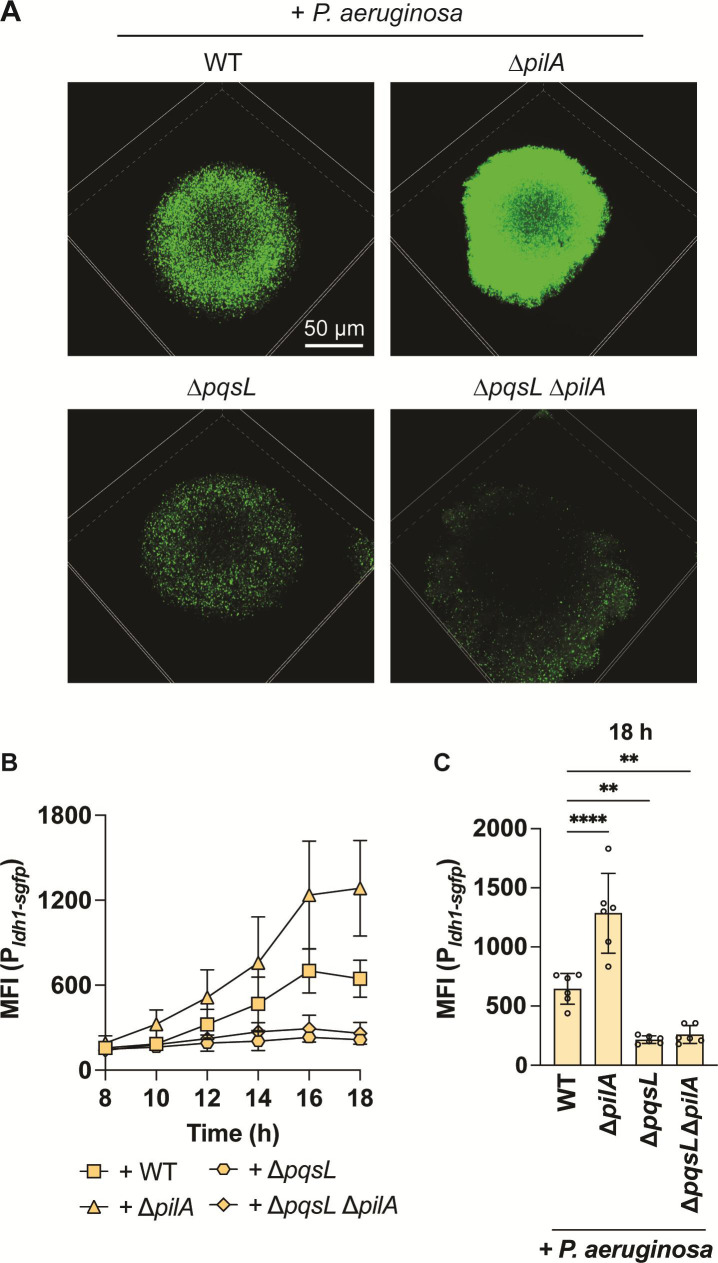
Increased *S. aureus* cell packing enhances HQNO-mediated *S. aureus* fermentation. *S. aureus* lactic acid fermentation (P*_ldh1_*_-*sgfp*_) was measured in the presence of the indicated *P. aeruginosa* strains. (A) Representative resonant scanning confocal micrographs of *S. aureus* fermentation in coculture with *P. aeruginosa* (WT, Δ*pilA*, Δ*pqsL*, or Δ*pqsL* Δ*pilA*) *t* = 18 hours, *S. aureus* channel only. (B) MFI (fluorescence/colony volume) was quantified over time. (C) MFI at 18 hours. Data represent the mean and standard deviation from three biological replicates with two technical replicates per condition. Each data point represents one technical replicate. Statistical analyses were performed at 18 hours using one-way ANOVA followed by Dunnett’s multiple comparisons test comparing each condition to +WT *P*. *aeruginosa*. **P < 0.01 and ****P < 0.0001.

Although *P. aeruginosa* antimicrobials did not influence *S. aureus* structure, we next explored how colony morphology differences affect *S. aureus* physiological response to HQNO by utilizing a fluorescent reporter system. HQNO poisons the *S. aureus* respiratory chain, forcing a shift to fermentative metabolism (11); therefore, *S. aureus* fermentation can be used as a proxy for HQNO activity. A fluorescent transcriptional fusion to the promoter of the lactate dehydrogenase gene (P*_ldh1_*_-*sgfp*_) was used to measure fermentation (47). If HQNO penetrates densely packed colonies, we expect to see increased fluorescence compared with coculture with *P. aeruginosa* lacking HQNO production. To test this prediction, we live imaged *S. aureus* in mono- or coculture with *P. aeruginosa* and quantified the mean fluorescence intensity (MFI) per *S. aureus* colony over 18 hours (Fig. 3). *S. aureus* P*_ldh1_*_-*sgfp*_ fluorescence began to increase at approximately 12 hours in coculture with WT *P. aeruginosa* but did not increase in ∆*pqsL* coculture, confirming prior reports that HQNO increases *ldh* expression (11). To test if fermentation increases in the absence of invasion, P*_ldh1_*_-*sgfp*_ expression was quantified in coculture with *P. aeruginosa* ∆*pilA*. Notably, we observed a sharp increase in fermentation of densely packed colonies produced by coculture with ∆*pilA* (shown in Fig. 3A; quantified in Fig. 3B and C). One interpretation of these data is that HQNO concentrates within densely packed colonies, inducing a more dramatic change in *S. aureus* physiology. Additionally, since ∆*pilA* cells grow around and against *S. aureus*, it is possible that the striking increase in *S. aureus* fermentation is due to more HQNO-producing cells surrounding *S. aureus* colonies, although precisely quantifying the cell number under these conditions is technically challenging. Alternatively, the increased P*_ldh1_*_-*sgfp*_ signal may result from increased cell density, independent of HQNO, potentially due to oxygen restriction within the colony. To differentiate these possibilities, fermentation was measured in the presence of a ∆*pqsL* ∆*pilA* mutant. As seen with motile ∆*pqsL*, the double mutant does not induce *S. aureus* fermentation over time (Fig. 3B), suggesting HQNO mediates this increased fermentation. Importantly, the phenotypes of both ∆*pilA* and ∆*pqsL* mutants were genetically complemented by expressing their respective genes in *cis* (∆*pilA*) or *trans* (∆*pqsL*) under control of an inducible promoter (Fig. S3). These findings show that HQNO likely diffuses into *S. aureus* colonies independently of *P. aeruginosa* invasion and plays a crucial role in mediating interspecies interactions by pushing *S. aureus* toward fermentation.

### *P. aeruginosa* TFP motility is necessary for competition against *S. aureus* in conditions that mimic CF lung secretions

So far, we see a role for TFP motility in competition against *S. aureus* under conditions that constrain cells to the surface. While useful for high spatial and temporal resolution, this approach does not accurately reflect other attributes of the CF airway infection environment. Thus, we sought to determine whether TFP motility drives interactions when cocultured under conditions that mimic CF lung secretions by using artificial sputum media (ASM) (5), a modified version of SCFM2 (48). ASM captures some of the essential features of the CF environment, like constraints on movement and diffusion, that shaken liquid culture methods do not, and similar recipes have been shown to recapitulate approximately 86% of *P. aeruginosa* gene expression in human-expectorated CF sputum, outperforming both laboratory media and the acute mouse pneumonia model of infection (49, 50).

**Fig 4:**
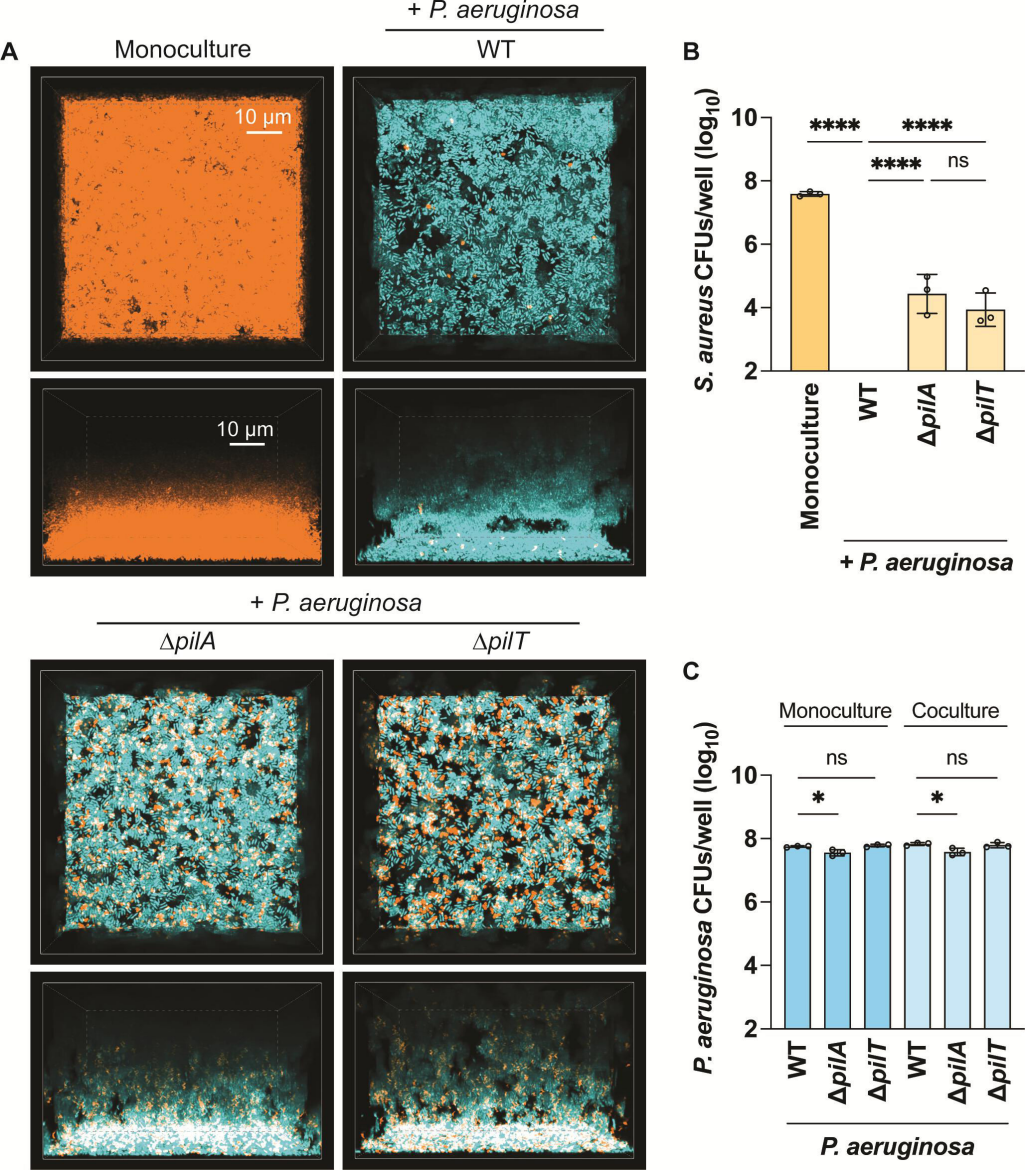
*P. aeruginosa* type IV pili motility is necessary for competition against *S. aureus* in artificial sputum media. Resonant scanning confocal imaging of *S. aureus* and *P. aeruginosa* under static conditions in artificial sputum media, with CFU quantification. (A) Representative images of resonant scanning confocal micrographs of WT *S. aureus* (pseudocolored orange) in monoculture or in coculture with *P. aeruginosa* (pseudocolored cyan; WT, Δ*pilA*, or Δ*pilT*) *t* ~ 22 hours. White indicates areas of overlap between *S. aureus* and *P. aeruginosa* suggesting colocalization. *S. aureus* (B) or *P. aeruginosa* (C) CFU quantification in monoculture or in coculture with *P. aeruginosa* (WT, Δ*pilA*, or Δ*pilT*) (B) or in mono- or coculture with *S. aureus* (C) at *t* = 24 hours. The CFUs/well in *Y*-axes are portrayed as log_10_ transformed. Three biological replicates with one technical replicate each were analyzed, and the mean and standard deviation are shown. Each data point represents one biological replicate. Statistical significance was determined by one-way ANOVA followed by Dunnett’s multiple comparisons test. n.s., not significant; *P < 0.05 and ****P < 0.0001.

*S. aureus* and *P. aeruginosa* (WT or ∆*pilA*) at a 1:1 ratio were grown statically for 22 hours and imaged with resonant scanning confocal microscopy to visualize their spatial organization. The end time point was plated for colony-forming units to assess bacterial viability. In the presence of WT motile *P. aeruginosa*, *S. aureus* was suppressed relative to its monoculture condition: very few *S. aureus* cells could be observed or counted (Fig. 4A and B), compared with approximately 10^8^ CFUs/well recovered in *S. aureus* monoculture. However, when *S. aureus* was grown with *P. aeruginosa* ∆*pilA*, a 100–10,000-fold increase in *S. aureus* cells was recovered in comparison to coculture with WT *P. aeruginosa* (Fig. 4B). Overall, this suggests that TFP motility is necessary for effective competition with *S. aureus* in ASM. TFP are also necessary for *P. aeruginosa* biofilm formation and attachment to surfaces (51–53). However, we observed that *P. aeruginosa* biofilm formation and spatial organization were similar in appearance between WT and ∆*pilA* in coculture with *S. aureus* in ASM (Fig. 4A). While the CFUs/well recovered for ∆*pilA* were significantly lower than WT *P. aeruginosa* in mono- or coculture with *S. aureus* (Fig. 4C), the difference is modest (~95% of WT) and thus not expected to account for the increase in *S. aureus* survival. Next, we tested if the mere presence of TFP has a role in competition or if TFP motility is required. To differentiate between these two outcomes, a hyperpiliated, non-twitching *P. aeruginosa* mutant (∆*pilT*) was cocultured with *S. aureus*. This mutant lacks the main retraction ATPase of the TFP machinery, PilT, and is well documented to ineffectively retract extended pili (54). *S. aureus* survival in the presence of ∆*pilT* phenocopied ∆*pilA* (Fig. 4A and B), suggesting that functional TFP are necessary for competitive interactions with *S. aureus* in ASM. Collectively, these data demonstrate that under CF-relevant conditions, *P. aeruginosa* TFP motility aids in interspecies competition.

### *P. aeruginosa* TFP motility is necessary for disruption and competition against pre-formed *S. aureus* biofilms

**Fig 5:**
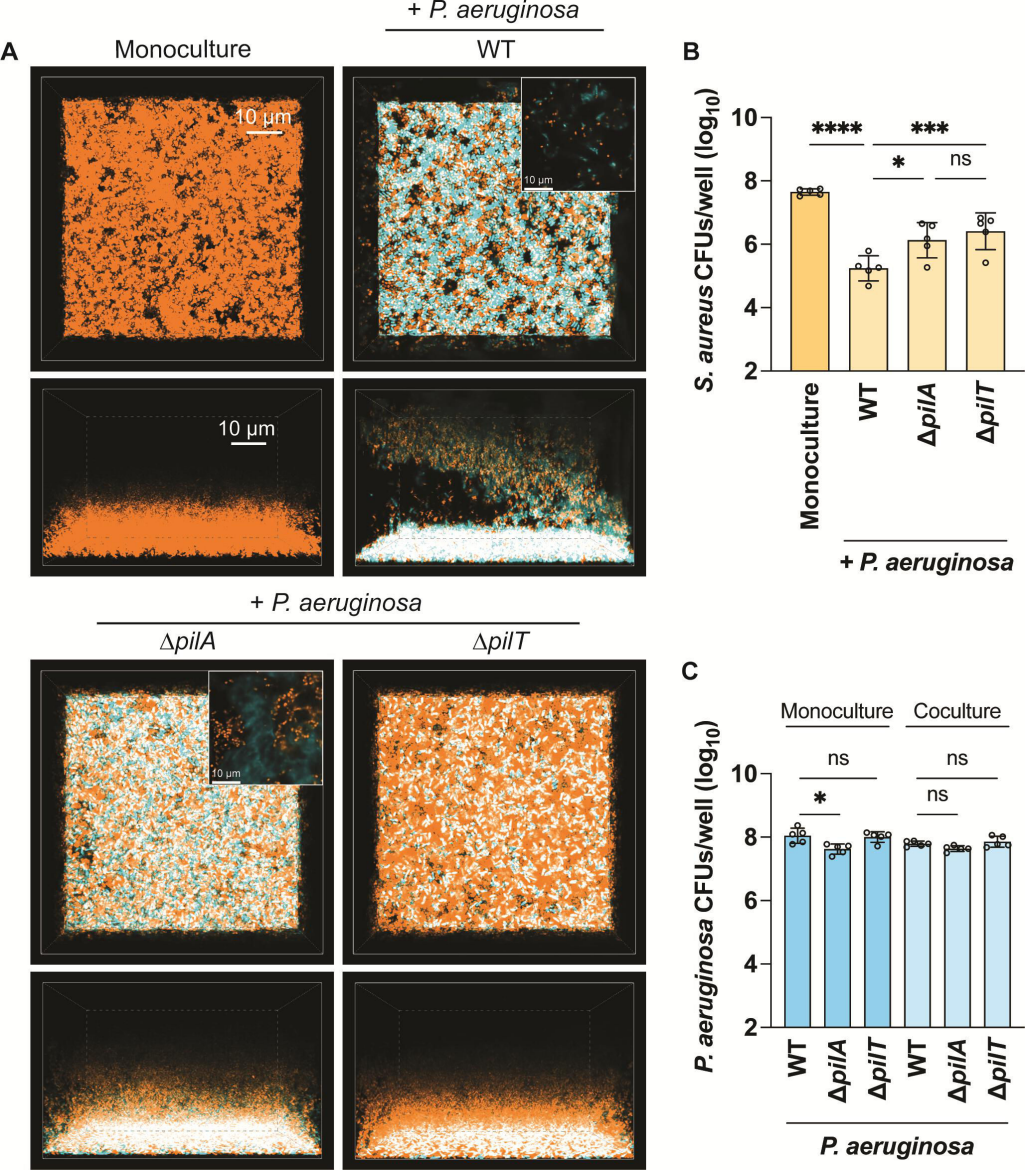
*P. aeruginosa* type IV pili motility is necessary for disruption and competition against pre-formed *S. aureus* biofilms. Resonant scanning confocal imaging of *S. aureus* and *P. aeruginosa* under static conditions in artificial sputum media, with CFU quantification at late time points. *P. aeruginosa* was added to *S. aureus* pre-formed biofilms at 5 hours. (A) Representative images of resonant scanning confocal micrographs of WT *S. aureus* (pseudocolored orange) in monoculture or in coculture with *P. aeruginosa* (pseudocolored cyan; WT, Δ*pilA*, or Δ*pilT*) *t* ~ 26 hours. The insets show ~3× zoomed images at 30 µm from the base of the coverslip. White indicates areas of overlap between *S. aureus* and *P. aeruginosa* suggesting colocalization. *S. aureus* (B) or *P. aeruginosa* (C) CFU quantification in mono- or in coculture with *P. aeruginosa* (WT, Δ*pilA*, or Δ*pilT*) (B) or in mono- or coculture with *S. aureus* (C) at *t* ~ 29 hours. The CFUs/well in *Y*-axes are portrayed as log_10_ transformed. Five biological replicates with one technical replicate each were analyzed, and the mean and standard deviation are shown. Statistical significance was determined by one-way ANOVA followed by Dunnett’s multiple comparisons test. n.s., not significant; *P < 0.05, ***P < 0.001, and ****P < 0.0001.

Next, we asked if *P. aeruginosa* TFP motility is necessary for competition against pre-formed (5 hours) *S. aureus* biofilms in ASM. *P. aeruginosa* WT, ∆*pilA*, or ∆*pilT* were added to *S. aureus* and allowed to grow for an additional 24 hours before imaging and plating for viability. Remarkably, we found that WT *P. aeruginosa* invades and disrupts pre-formed *S. aureus* biofilms, as depicted in Fig. 5A (Z plane) and Movie S1, where a layer of the *S. aureus* biofilm detaches from the surface and is blanketed by *P. aeruginosa* cells. Disruption was dependent on *P. aeruginosa* TFP motility, as WT *P. aeruginosa* disrupted *S. aureus* pre-formed biofilms significantly more than ∆*pilA* or ∆*pilT* (Fig. S4). Notably, *S. aureus* and *P. aeruginosa* ∆*pilA* remained segregated into monoculture aggregates, while cells were well mixed during coculture with motile *P. aeruginosa* (Fig. 5A, inset). These observations were consistent with results under agarose pads. We also observed that a higher number of WT *P. aeruginosa* colonized the surface of the coverslip compared with the ∆*pilA* or ∆*pilT* mutants in the presence of *S. aureus* (Fig. 5A; Fig. S4). These data suggest that TFP motility is necessary for *P. aeruginosa* cells to invade from the top of *S. aureus* biofilms and traverse through to access the coverslip, potentially disrupting and detaching the biofilms in the process. This results in a significant reduction in *S. aureus* viability (10–15-fold) when comparing *S. aureus* coculture with WT versus ∆*pilA* or ∆*pilT* (Fig. 5B). Importantly, no viability differences were observed between *P. aeruginosa* WT and ∆*pilT* (Fig. 5C). While ∆*pilA* shows a significant decrease in viability compared with WT, it is unlikely to have a biological influence on *S. aureus* growth (Fig. 5C). Altogether, these observations suggest that TFP motility enhances *P. aeruginosa* competitive fitness, allowing it to disrupt and potentially render *S. aureus* cells more vulnerable to *P. aeruginosa* antimicrobials.

## DISCUSSION

Growing data support the hypothesis that spatial organization is crucial in shaping microbial communities and influencing community-based behaviors (4, 5, 39, 55–61). In this study, we found that *P. aeruginosa* motility plays a vital role in shaping the biogeography in *S. aureus* cocultures. By influencing spatial aggregation, *P. aeruginosa* TFP motility ultimately dictates *S. aureus* physiology and survival.

While *P. aeruginosa* antimicrobials have been well documented to influence *S. aureus* growth and survival (11–16), *P. aeruginosa* motility in interspecies competition has only begun to be explored. We recently reported that *P. aeruginosa* senses *S. aureus*-secreted PSM peptides from a distance by the PilJ chemoreceptor (43). Consequently, it employs TFP motility to chemotax toward *S. aureus* colonies or PSMs alone (42, 44). In addition to chemotaxis, *S. aureus* PSMs also trigger a “competition sensing” response whereby *P. aeruginosa* upregulates type VI secretion system and pyoverdine biosynthesis pathways (44). Similarly, *P. aeruginosa* has been reported to utilize TFP-mediated motility to perform “suicidal chemotaxis” toward antibiotics (62). The upregulation of these common interbacterial competition pathways supports a model where *P. aeruginosa* senses potential danger in the environment and responds with directional twitching, while simultaneously activating defense mechanisms to combat the “enemy”. Additionally, it has been reported that *P. aeruginosa* upregulates antimicrobial production upon sensing *N*-acetylglucosamine alone or shed from Gram-positive bacteria (63).

**Fig 6:**
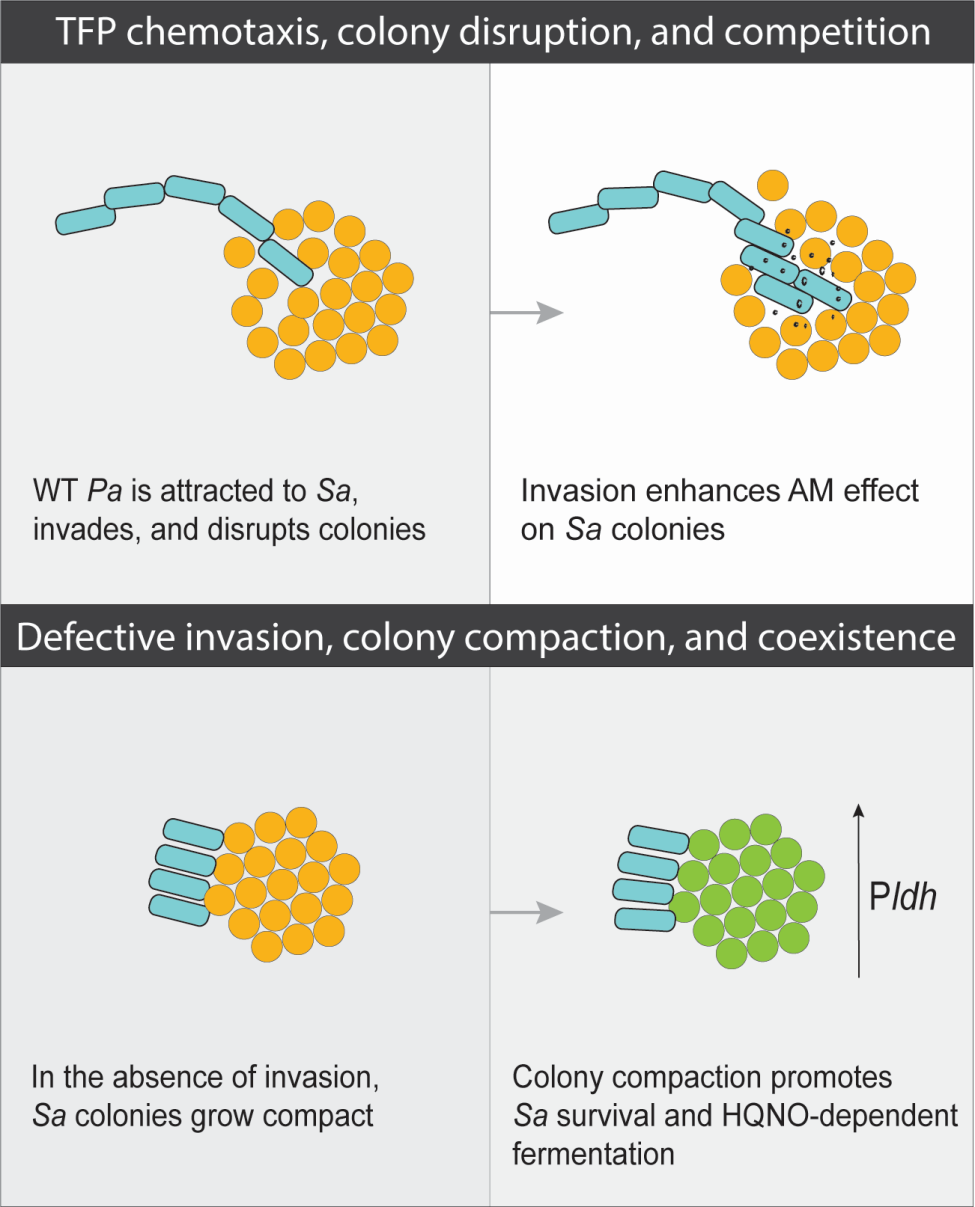
Model for motility-driven interspecies competition. We propose that *P. aeruginosa* (*Pa*) TFP motility-mediated attraction toward, invasion, and disruption of *S. aureus* (*Sa*) colonies promote the diffusion of antimicrobials (AM) to maximize interspecies competition. Lack of motility affects *S. aureus* spatial organization and physiology in a manner that promotes coexistence.

Our single-cell level temporal analysis also revealed that *P. aeruginosa* TFP motility is necessary for invading and disrupting *S. aureus* colonies (Fig. 1). Interestingly, loss of invasion leads *P. aeruginosa* to grow adjacent to *S. aureus* colonies, potentially acting as a “wall” to prevent expansion of the *S. aureus* colonies, which become thicker and denser (Fig. 2). While *P. aeruginosa* anti-staphylococcal factors did not mediate invasion into *S. aureus* colonies, they did influence growth as *S. aureus* formed larger colonies in the absence of *P. aeruginosa* antimicrobials HQNO, pyoverdine, pyochelin, and staphylolysin (Fig. 2B), as expected based on prior reports (11, 13, 16). However, most studies have been performed with *P. aeruginosa* cell-free supernatant and not with live *P. aeruginosa* present. Imaging *P. aeruginosa* and *S. aureus* in coculture at the single-cell level has allowed us to visualize the importance of *P. aeruginosa* motility in their interactions and, therefore, start to build a model whereby TFP motility aids in competition by disrupting *S. aureus* single cells away from the colony, leaving them exposed and more vulnerable to *P. aeruginosa*-secreted factors (Fig. 6). Therefore, when *P. aeruginosa* cannot move, we hypothesize that *S. aureus* cells remain protected within the colony and resist infiltration of *P. aeruginosa* antimicrobials. Altogether, these findings provide additional support of how TFP motility can either enhance competition or foster coexistence with *S. aureus*.

Different *S. aureus* colony morphology is a consequence of limited invasion by *P. aeruginosa* ∆*pilA*, compared with WT, and shows a striking change in physiology by increasing fermentation (P*_ldh1_*_-*sgfp*_) (Fig. 3). While we initially hypothesized that *S. aureus* cells remained protected from *P. aeruginosa* antimicrobials in the absence of invasion, these data suggest that HQNO can diffuse into *S. aureus* colonies and alter growth and physiology without *P. aeruginosa* TFP-mediated invasion. Nevertheless, deletion of antimicrobial production in the ∆*pilA* mutant background does not significantly improve *S. aureus* survival, as it does in the WT background. This observation supports the role of TFP-mediated invasion and disruption in allowing these antimicrobials to access *S. aureus* cells within the colonies for greater impact on its growth (Fig. 2B). Therefore, we hypothesize that without invasion and disruption, antimicrobials with higher molecular weight, such as staphylolysin (20 kDa), are precluded from freely diffusing into *S. aureus* colonies, while smaller compounds like HQNO (0.259 kDa) can diffuse and concentrate within *S. aureus*, eliciting physiological changes that could pose greater challenges for the effective treatment of infections.

Importantly, *P. aeruginosa* TFP motility’s role in mediating interspecies competition and spatial aggregation was highlighted under conditions that mimic the nutritional and viscoelastic properties of CF airways. Of note, *P. aeruginosa* was capable of detaching preformed *S. aureus* biofilms and significantly reducing *S. aureus* viability in a motility-dependent manner when grown in ASM. These results emphasize the importance of motility in this CF-like polymicrobial environment.

The mucoid *P. aeruginosa* phenotype, a common adaptation that *P. aeruginosa* exhibits during CF infections, is associated with decreased competition against *S. aureus* due to the reduced production of anti-staphylococcal factors (46). Additional studies have demonstrated that another common adaptation of *P. aeruginosa* linked to chronic CF infections is reduced motility (64). Interestingly, *P. aeruginosa* mucoid and reduced twitching phenotypes have been identified as the best phenotypic predictors of future pulmonary exacerbations in children with CF (64). Our studies revealed that impairing twitching motility hinders *P. aeruginosa* competitiveness and promotes coexistence with *S. aureus* under CF-relevant conditions. This may be a contributing explanation for why *P. aeruginosa* and *S. aureus* are still found in high numbers in people with CF (18).

Extensive discussion surrounds whether *P. aeruginosa* and *S. aureus* encounter each other in CF lungs and if they compete or coexist (4, 39, 46, 55). CF airways are indeed a complex environment with multiple, distinct niches within; therefore, we predict that *P. aeruginosa* and *S. aureus* can coexist in some areas, while *P. aeruginosa* might outcompete *S. aureus* in others. They may be well mixed in some spaces and spatially segregated in others but still influence each other through the diffusion of secreted factors. As evidenced by both this study and others, it is clear that interspecies competition or coexistence can greatly depend on bacterial and host genotype and phenotype. Our *in vitro* data show how spatial organization can determine the outcome of microbe-microbe interactions and inform the potential interactions of these bacteria during infection. However, the CF airways are complex and involve other microorganisms and host factors that must be considered. Therefore, future experiments should explore these interactions *in vivo* and *ex vivo* to map the community biogeography and further elucidate interspecies dynamics.

Overall, our study reveals how *P. aeruginosa* TFP motility aids in the disruption of *S. aureus* colonies and biofilms, which potentiates the effect of *P. aeruginosa*-secreted anti-staphylococcal factors on *S. aureus* cells. Ultimately, *P. aeruginosa* motility plays a crucial and previously unexplored role in determining *S. aureus* outcome.

## MATERIALS AND METHODS

For additional details on all the methods, see the supplemental materials and methods in Text S1.

### Bacterial strains and growth conditions

See Text S1 for details on bacterial growth conditions. A list of strains used in this study can be found in Table S1.

### Generation of *P. aeruginosa* deletion mutants

Markerless deletion mutants of genes in PA14 were constructed through homologous recombination as previously described (65).

### Time-lapse microscopy

*P. aeruginosa* and *S. aureus* were cocultured under agarose pads as previously described (42) and live imaged with resonant scanning confocal microscopy.

### *S. aureus* colony edge height and density measurements

*P. aeruginosa* and *S. aureus* were cocultured under agarose pads for ~24 hours and imaged with galvanometer scanning confocal microscopy. *S. aureus* colony edge height and density were analyzed in Nikon Elements and BiofilmQ (45), respectively.

### *S. aureus* lactic acid fermentation (P*_ldh1_*_-s*gfp*_) quantification

*P. aeruginosa* and *S. aureus* were cocultured under agarose pads and live imaged with resonant scanning confocal microscopy. *S. aureus* lactic acid fermentation was quantified by measuring the mean fluorescence intensity (P*_ldh1_*_-s*gfp*_) of whole colonies over time.

### Artificial sputum media assay

*P. aeruginosa* and *S. aureus* were cocultured in ASM for ~24 or 29 hours and imaged with resonant scanning confocal microscopy. ASM was prepared as previously described (5).

### *S. aureus* lysis assay and growth curve with *P. aeruginosa* supernatant

*S. aureus* lysis in the presence of *P. aeruginosa* cell-free supernatant was assessed by modifying a previously published method (66). *S. aureus* growth with *P. aeruginosa* supernatant was assessed in a plate reader for 18 hours.

## SUPPLEMENTAL MATERIAL

Text S1 mbio.00956-24-s0001.pdf Supplemental materials and methods.

Figure S1 mbio.00956-24-s0002.tif *P. aeruginosa* cells surround *S. aureus* colonies at the end time point.

Figure S2 mbio.00956-24-s0003.tif Exoproducts from Δ*pilA* lyse and inhibit *S. aureus* to the same levels as WT *P. aeruginosa* factors.

Figure S3 mbio.00956-24-s0004.tif Genetic complementation of *pqsL* or *pilA*.

Figure S4 mbio.00956-24-s0005.tif *P. aeruginosa* type IV pilus motility is necessary for disrupting pre-formed *S. aureus* biofilms.

Supplemental Figures mbio.00956-24-s0006.pdf Supplemental figures.

Supplemental Tables mbio.00956-24-s0007.pdf Tables S1 to S3.

Movie S1 mbio.00956-24-s0008.mov WT *P. aeruginosa* (cyan) disrupts *S. aureus* (orange) biofilms in ASM. Movie is at 29 hours.

ASM does not own the copyrights to Supplemental Material that may be linked to, or accessed through, an article. The authors have granted ASM a non-exclusive, world-wide license to publish the Supplemental Material files. Please contact the corresponding author directly for reuse.
