# Supplementary material for: Pseudomonas aeruginosa surface motility and invasion into competing communities enhance interspecies antagonism
Source: mBio. 2024 Aug 6;15(9):e00956-24. doi: 10.1128/mbio.00956-24 (PMC11389416; doi:10.1128/mbio.00956-24)
Supplement: Text S1 — Supplemental materials and methods. [file mbio.00956-24-s0001.pdf]

SUPPLEMENTARY TEXT

Supplemental Materials and Methods

Bacterial strains and growth conditions. For microscopy assays, *P. aeruginosa* (PA14) and *S. aureus* (USA300 JE2) were grown in M8T medium (M8 salts supplemented with 0.2% glucose, 1.2% tryptone, and 1 mM $MgSO_4$). *S. aureus* fluorescent reporter plasmids were maintained with 7.5 µg/mL (pCM29) or 10 µg/mL (pEM87) chloramphenicol. For all other experiments, *P. aeruginosa* and *Escherichia coli* were grown in lysogeny broth (LB), and *S. aureus* in tryptic soy broth (TSB; BD Bacto^{TM}), unless otherwise specified. Bacteria were grown overnight rotating at 30°C (*E. coli* with pMQ30 background vector) or 37°C for 14 to 16 hours. Subcultures were also grown rotating at their respective temperatures.

Generation of *P. aeruginosa* deletion mutants. To generate the Δ*lasA* mutants, ~800 bp upstream and downstream of *lasA* were amplified from WT *P. aeruginosa* PA14 chromosome with primers ASP12_lasA_KO_HindIII_UP_F and ASP13_lasA_KO_UP_R (upstream) and ASP14_lasA_KO_DN_F and ASP15_lasA_KO_SacI_DN_R (downstream) (Table S3) and cloned into pEXG2-Tc with Gibson assembly (New England Biolabs). pEXG2-Tc-Δ*lasA* (Table S2) was introduced into *E. coli* S17 and conjugated with *P. aeruginosa* to perform two-step allelic exchange, and counter selected with 10% sucrose. The Δ*pilA* deletion mutants were generated with the pSMC259-Δ*pilA* (Table S2) following the same protocol. Deletion mutants were confirmed by PCR with primers ASP18_lasA_KO_seq_Fwd and ASP19_lasA_KO_seq_Rev for Δ*lasA*, and oDHL34_pilA-check-F and oDHL35_pilA-check-R for Δ*pilA* (Table S3) for the regions flanking the target genes, followed by Sanger sequencing of the PCR products.

Integration of mini-Tn7-*mCherry* into *P. aeruginosa* chromosome. The mini-Tn7 element was integrated into *P. aeruginosa att*Tn7 site following a modified protocol from Choi & Schweizer (2006) (1). *P. aeruginosa* overnights were grown in TSB and made electrocompetent with 300 mM sucrose. 300 - 500 ng of pBT277 and the helper plasmid pTNS3 each were simultaneously electroporated into *P. aeruginosa* target strains (not exceeding 5 µL total). To recover, cells were incubated rotating at 37°C for 1.5 hours and then plated on TSB agar with 30 µg/mL gentamicin, and left incubating overnight at 37°C.

Time-lapse microscopy. *P. aeruginosa*-mCherry strains and *S. aureus* pCM29 were grown overnight in M8T and subcultured in fresh M8T (no antibiotic selection) until mid-exponential phase (OD_{600} of ~0.3 - 0.8). *P. aeruginosa* and *S. aureus* were standardized to OD_{600} of 0.010 and 0.020, respectively, in pre-warmed M8T. Agarose pads were prepared by pipetting 550 µL of M8T with 2% molten agarose (Lonza, Cat. no. 50081) into each quadrant of a 4-chamber glass-bottom dish (Cellvis, Cat. no. D35C4-30-1.5-N) and drying uncovered for ~1.5 hours at room temperature, followed by ~1 hour covered with a lid at room temperature, then ~1.5 hours at 37°C. The standardized *P. aeruginosa* and *S. aureus* were mixed 1:1 in a 1.5 mL tube, vortexed, and 0.4 µL were added to one quadrant of a 4-chamber glass-bottom dish before transferring the pads onto the inoculated glass-bottom dish. For monoculture conditions, *S. aureus* or *P. aeruginosa* were mixed with M8T 1:1. Fields of view with *S. aureus* and *P. aeruginosa* single cells positioned approximately 30 to 50 µm apart were selected. Resonant scanning confocal microscopy was employed with a Nikon Eclipse Ti2 A1R and a 60x Plan Apo λ oil objective (1.4 NA). Two Z-stacks (40 - 60 µm) with a 0.12 µm step size, at different XY positions per condition were acquired at 1-hour intervals for 18 hours, followed by an end time point at ~24 hours. A 488 nm laser (laser power between 3 - 5%, offset -15, and HV – gain between 15 - 25) was used to excite the sGFP produced by *S. aureus* cells, and a 561 nm laser (laser power between 8 - 10%, offset 0, and HV – gain 45) to excite the mCherry in *P. aeruginosa* strains, with a pinhole set between 30 and 39 µm. The phase contrast images were acquired with an Andor Sona camera using a 100x Plan Apo λ oil Ph3 objective (1.45 NA) with 1.5x zoom. All images were saved and analyzed with the Nikon NIS-Elements AR Software.

***S. aureus* colony edge height and density measurements.** *S. aureus* pCM29 and *P. aeruginosa*-mCherry were grown overnight and subcultured in M8T and prepared for imaging in 4-chamber dishes as described above. At approximately 24 hours, galvanometer scanning confocal microscopy was employed to image 30 µm Z-stacks (0.12 µm step size) of *S. aureus* colony edges with a 60x Plan Apo λ oil objective (1.4

NA) with 1.5x zoom. The 488 nm laser (laser power 4%, offset -15, and HV – gain 20) with a pinhole of 35 μm was used to excite *S. aureus* GFP cells.

A vertical cross-section crop 15 μm into the colony was performed, and the height on the Z-plane was assessed using the measuring tool for volume projections in Nikon Elements.

The density of *S. aureus* was determined with the BiofilmQ framework, by quantifying the biofilm surface roughness and the architecture local density (cell packing) parameters. Segmented microbial volumes were divided into a 3D grid with each node 0.72 μm on a side. Neighborhood cell packing measurements calculated the local biovolume fraction within 6 μm of each segmented bacterial volume within each grid cube. Biofilm surface roughness was calculated by measuring the surface area within 4 μm around each grid cube. Each pixel within the heatmaps (Fig. 2D and E) shows the average roughness or cell packing value at every height in the Z-stack at that specific XY coordinate.

S. aureus **lactic acid fermentation ($P_{ldh1\text{-}sgfp}$) quantification.** *P. aeruginosa*-mCherry and *S. aureus* pEM87 were grown overnight in M8T. Overnight cultures were subcultured in fresh M8T with 10 μg/mL chloramphenicol used for *S. aureus*. Bacteria were then prepared for imaging under agarose pads as described above. Time-lapse resonant scanning confocal microscopy was performed as described above with a 60x Plan Apo λ oil objective (1.4 NA), for a total of 18 hours with 1-hour intervals. A 488 nm laser (laser power 6%, offset -20, and HV – gain 30) was used to excite the sGFP in *S. aureus* cells, and a 561 nm laser (laser power 6%, offset 0, and HV – gain 45) to excite the mCherry in *P. aeruginosa* strains, with a pinhole of 39.6 μm.

The Mean Fluorescence Intensity (MFI) from *S. aureus* colonies was measured in the Nikon Elements software using the 3D Measurements tool. This measurement takes into consideration the volume of the colony and is calculated with the following equation: $Mean\ Intensity = \frac{\frac{SumInt}{Volume}}{Voxel\ Volume}$. The threshold used for *S. aureus* colonies was the following: 150 - 4095 for brighter colonies (*S. aureus* in coculture with WT or Δ*pilA P. aeruginosa*) or 50 - 4095 for dimmer colonies (*S. aureus* in coculture with Δ*pqsL* or Δ*pqsL* Δ*pilA P. aeruginosa*).

Arabinose-inducible genetic complementation of *pqsL* and *pilA*. *S. aureus* pEM87 and *P. aeruginosa* strains were grown overnight in M8T. *P. aeruginosa* strains harboring the pMQ72-P_{araBAD} empty vector or pMQ72-$P_{araBAD-pqsL}$ (Table S2) were cultured with 30 µg/mL gentamicin in the overnights and subcultures. No antibiotic selection was used for the Δ*pilA* complementation strains which contain the chromosomal *att*Tn7 arabinose-inducible system (Table S1). Strains were subcultured in M8T until mid-exponential phase with 0.4% arabinose in the *P. aeruginosa* cultures. Cells were then prepared, imaged, and analyzed as described above using the same settings as the previous section (*S. aureus* $P_{ldh1-sgfp}$), with the addition of 0.4% arabinose to the agarose pads to induce $P_{araBAD-pqsL}$ or $_{-pilA}$.

Artificial sputum media assay. *P. aeruginosa*-mCherry and *S. aureus* pCM29 were grown overnight in M8T. The overnight liquid cultures were subcultured in fresh M8T (no antibiotic selection) and grown rotating until mid-exponential phase. Cultures were normalized to an OD_{600} of 0.2 in M8T and then washed 1X in PBS. The washed cultures were then inoculated into artificial sputum media at an OD_{600} of 0.025 in mono- or coculture in a total volume of 500 µL in a 1.5 mL microcentrifuge tube. The tubes were mixed by vortexing, and then 200 µL were inoculated into each quadrant of a 4-chamber glass-bottom 35 mm dish. The bacteria were grown statically for ~24 hours inside the microscope's incubation chamber at 37°C with 90% relative humidity controlled by the Okolab Humidity Controller. At ~22 hours, two 50 µm Z-stacks at different XY positions per condition were acquired with an inverted Nikon Eclipse Ti2 A1R Resonant Scanning Confocal Microscope, using a CFI SR HP Plan Apochromat Lambda S 100XC Sil objective (1.35 NA) with a 56.19 µm pinhole and 1024 x 1024 pixels. A 488 nm laser (laser power 5, offset -10, and HV – gain 10) was used to excite the sGFP in *S. aureus* cells, and a 561 nm laser (laser power 7, offset -10, and HV – gain 40) to excite the mCherry in *P. aeruginosa* strains.

For the experiments where *P. aeruginosa* was added to *S. aureus* pre-formed biofilms, *S. aureus* was prepared the same way as described above and incubated statically in monoculture for 4 - 5 hours. *P. aeruginosa* strains at a final concentration of

OD_{600} 0.03 were then added to *S. aureus* and incubated for an additional 24 hours. Then, 50 µm image stacks were acquired at ~29 hours.

The images were saved and analyzed with the Nikon Elements software. The representative images in Figs. 4 and 5 were deconvolved and are projected as a volume view with the Maximum Intensity Projection blending.

S. aureus and *P. aeruginosa* growth was assessed at ~24 hours (when *P. aeruginosa* was coinoculated with *S. aureus* at T0) or ~29 hours (when *P. aeruginosa* was added to pre-formed *S. aureus* biofilms) by plating the serial dilutions on selective media (Mannitol Salt Agar (MSA) and *Pseudomonas* Isolation Agar (PIA), respectively). To harvest the bacteria from the microscopy dishes, 0.2% Triton X-100 was added to the cultures and incubated at room temperature for 10 - 20 m. Then, the bacteria were scrapped with a pipette tip and all the volume per quadrant was transferred to a 1.5 mL centrifuge tube. The tubes were thoroughly mixed by vortexing prior to performing the serial dilutions.

***S. aureus* cell disruption measurement.** The end-time point Z-stack images from the artificial sputum data (Fig. 5 and Supplementary Fig. 4) were analyzed in the Nikon Elements analysis software. Using the 3D Measurements tool, the volume of the bottom four Z planes (i.e., *S. aureus* cells attached to the coverslip) was divided by the volume of the rest (top part) of the *S. aureus* biofilm.

***P. aeruginosa* supernatant collection.** *P. aeruginosa* strains were grown overnight in LB. The cultures were standardized to OD_{600} 3.0 in LB and spun for 5 m at 15 krpm. The supernatants were filter sterilized with a PES 0.22 µm filter.

***S. aureus* lysis assay with *P. aeruginosa* supernatant.** *S. aureus* and *P. aeruginosa* were grown overnight in TSB or LB, respectively. *P. aeruginosa* cell-free supernatants were collected as described above. *S. aureus* was subcultured in TSB starting at OD_{600} 0.1 and grown for 3 hours. All the volume from the cultures (5 mL) was centrifuged for 10 m at 4 krpm, washed twice in the same volume of cold (4°C) sterile distilled water, and resuspended in buffer (50 mM Tris-HCl (pH 7.2) with 0.05% Triton X-100). The bacteria

were then standardized to OD_{600} 1.0 in buffer and 500 µL of *P. aeruginosa* supernatant (OD_{600} 3.0) were added to triplicate plastic cuvettes followed by 500 µL of *S. aureus* in buffer (OD_{600} 1.0). For the *S. aureus* control, 500 µL of buffer plus 500 µL of *S. aureus* cells were used. Cuvettes were covered with parafilm, mixed by inversion, and the initial OD_{580} measurement was acquired with a spectrophotometer. Then, the samples were incubated shaking (225 rpm) at 30°C, and measurements were taken every 30 m for a total of 2.5 hours.

***S. aureus* growth curve with *P. aeruginosa* supernatant.** *S. aureus* and *P. aeruginosa* were grown overnight in TSB or LB, respectively. *S. aureus* was subcultured in TSB until mid-exponential phase. *P. aeruginosa* supernatants were collected as described above. *S. aureus* cells were normalized to OD_{600} 0.02 in TSB and mixed 1:1 with *P. aeruginosa* supernatant (collected from cultures at OD_{600} 3.0) in a 1.5 mL microcentrifuge tube, for a final concentration of *S. aureus* at OD_{600} 0.01 and *P. aeruginosa* supernatant at OD_{600} 1.5. For monoculture *S. aureus*, cells were mixed 1:1 with LB, since the *P. aeruginosa* supernatants were derived from cultures grown in LB. The tubes were thoroughly mixed, and 200 µL were added to triplicate wells of a clear flat bottom 96-well plate. The plate was covered with a gas-permeable membrane and OD_{600} measurements were taken every 15 m on a Tecan infinite M200 plate reader for 18 hours total. The plate was incubated statically at 37°C and was shaken for 30 s before each measurement.

SUPPLEMENTARY TEXT REFERENCES

1. Choi KH, Schweizer HP. 2006. mini-Tn7 insertion in bacteria with single *att*Tn7 sites: Example *Pseudomonas aeruginosa*. Nat Protoc 1:153-61.
