# Supplementary figures and images for: Pseudomonas aeruginosa surface motility and invasion into competing communities enhance interspecies antagonism
Source: mBio. 2024 Aug 6;15(9):e00956-24. doi: 10.1128/mbio.00956-24 (PMC11389416; doi:10.1128/mbio.00956-24)

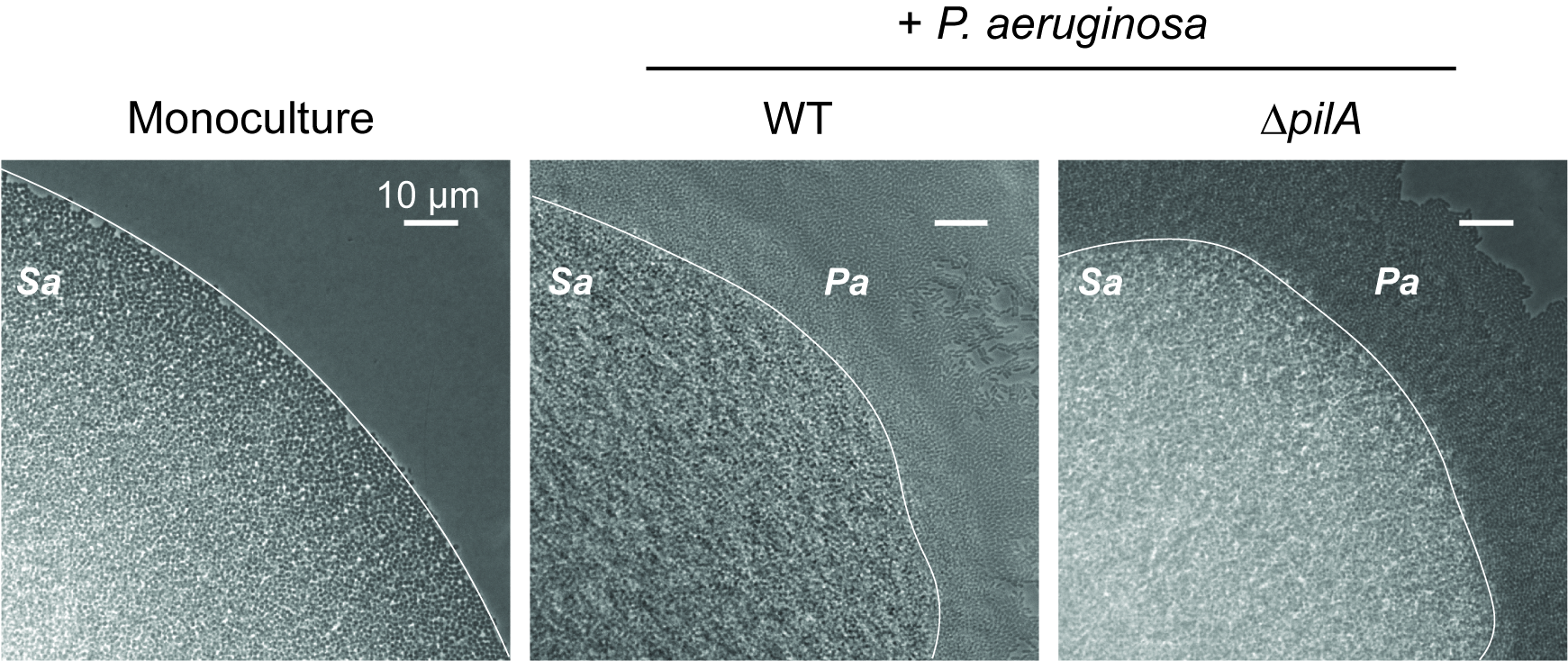

Supplement: Figure S1 — P. aeruginosa cells surround S. aureus colonies at the end time point. [file mbio.00956-24-s0002.tif]

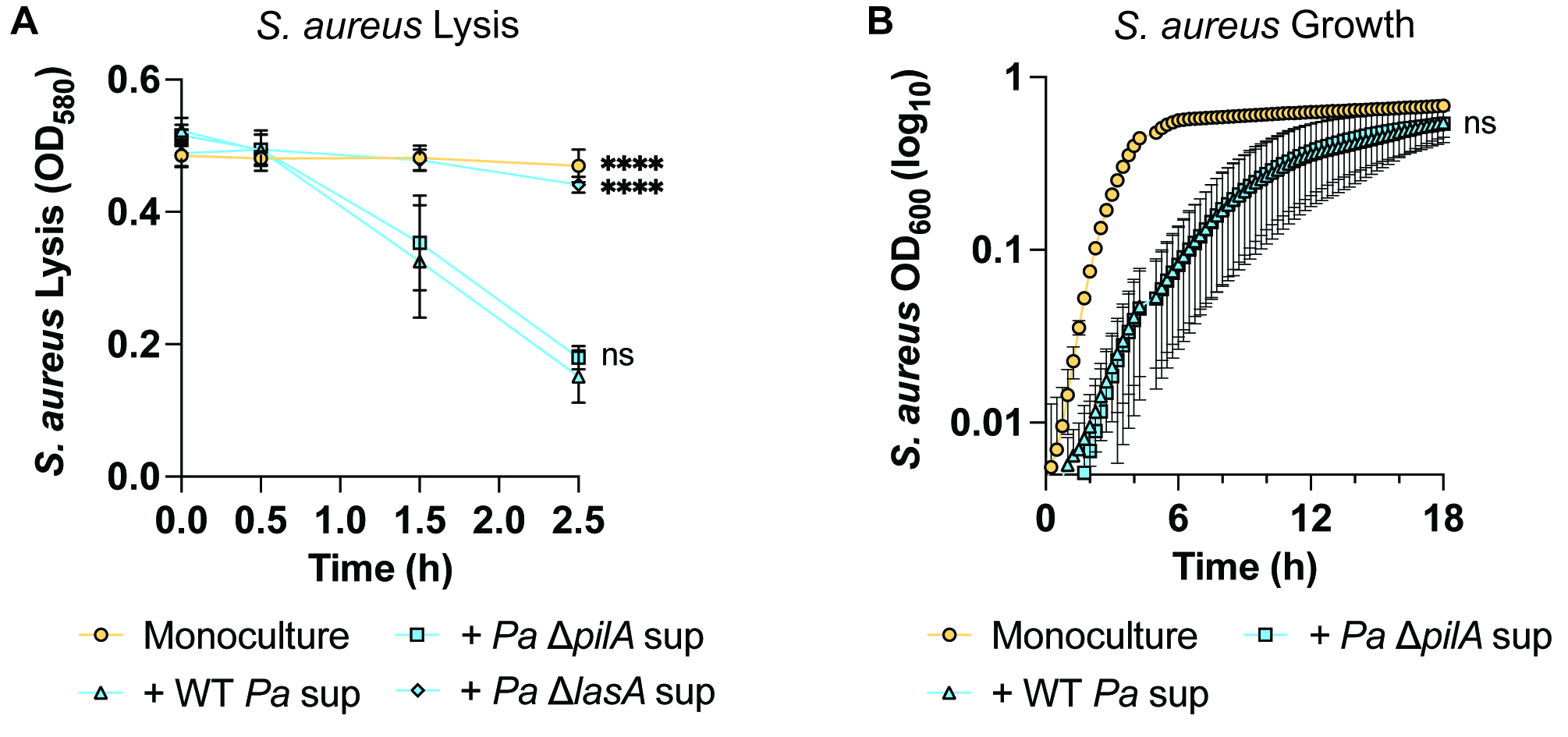

Supplement: Figure S2 — Exoproducts from ΔpilA lyse and inhibit S. aureus to the same levels as WT P. aeruginosa factors. [file mbio.00956-24-s0003.tif]

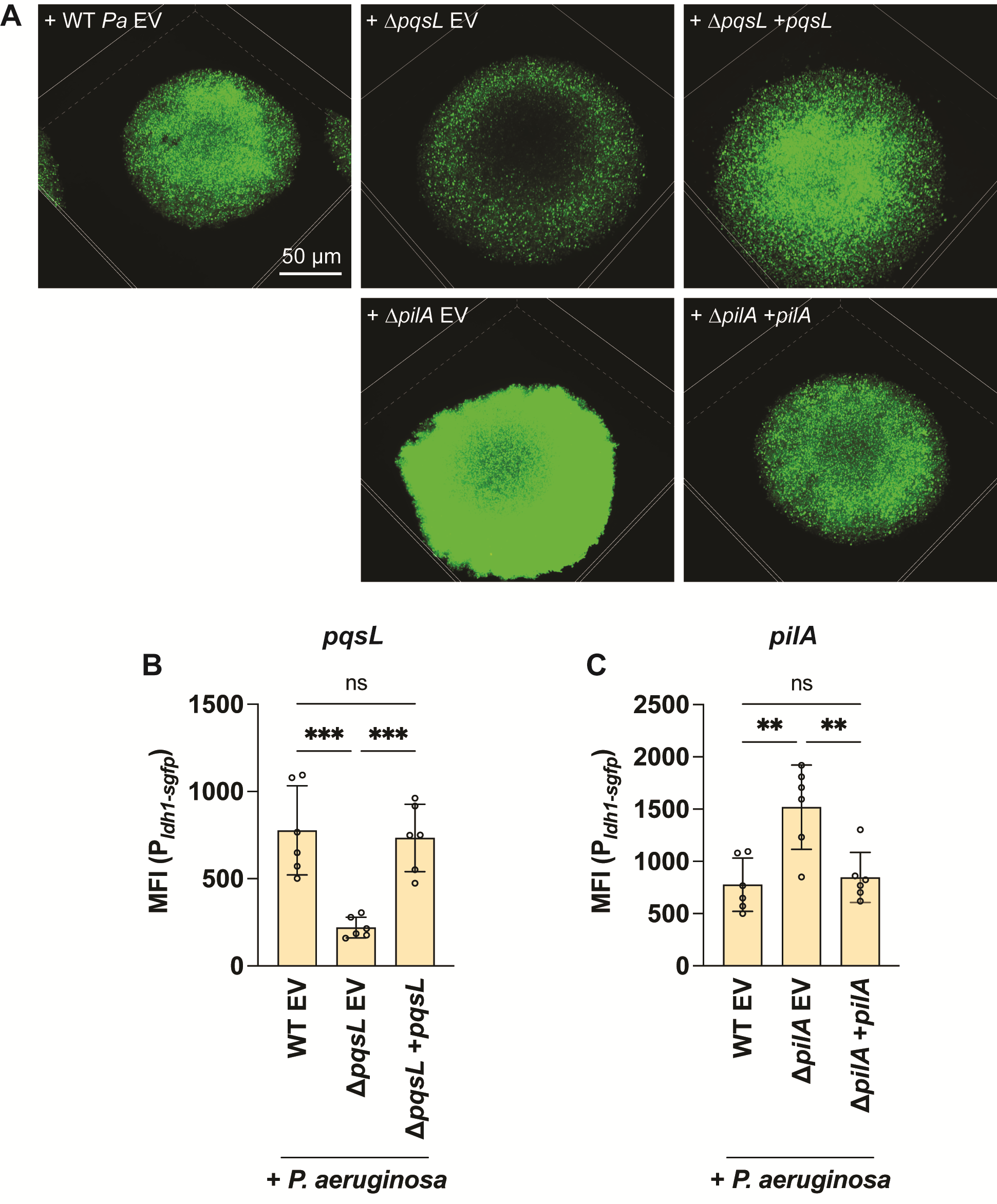

Supplement: Figure S3 — Genetic complementation of pqsL or pilA. [file mbio.00956-24-s0004.tif]

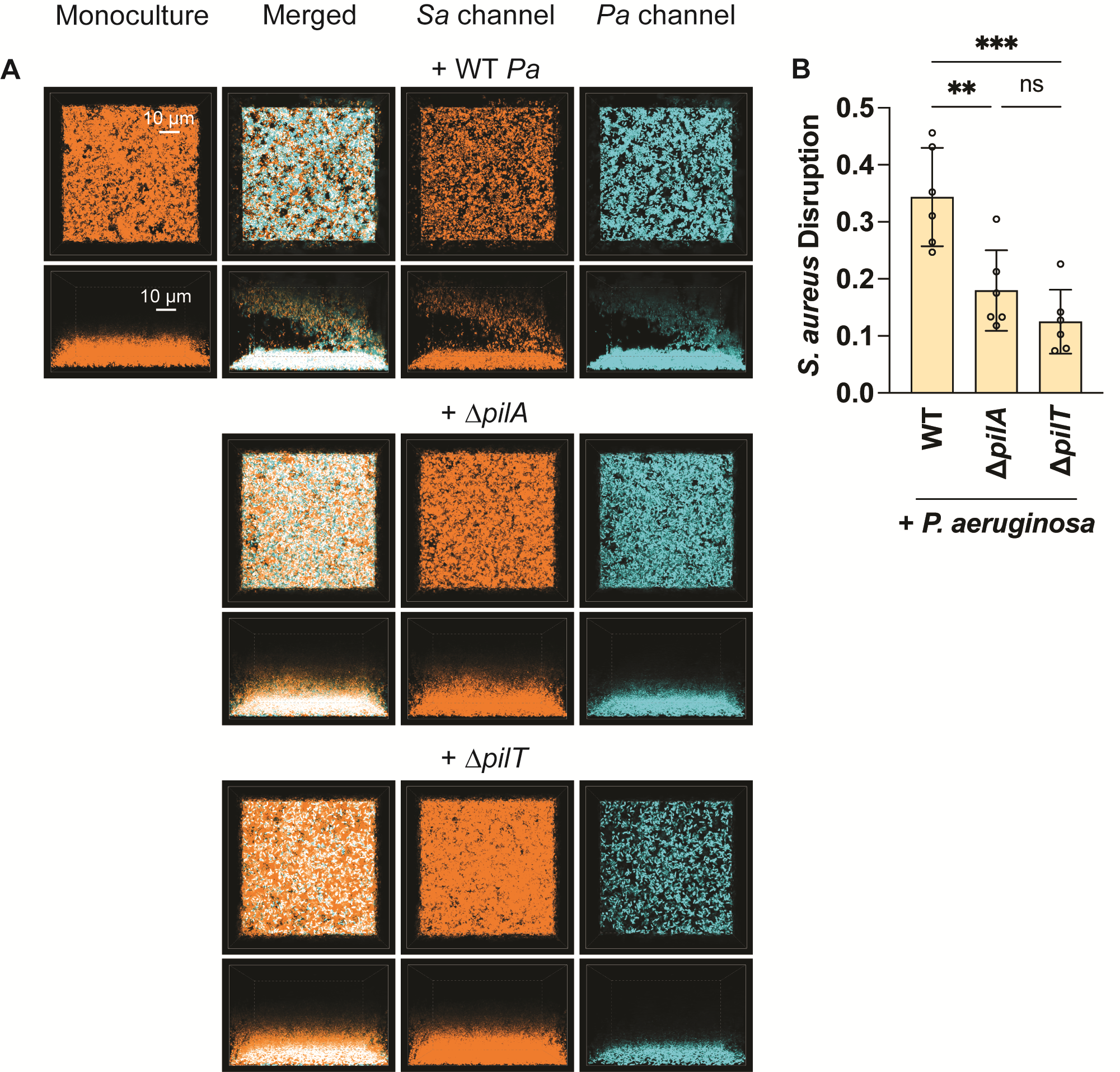

Supplement: Figure S4 — P. aeruginosa type IV pilus motility is necessary for disrupting pre-formed S. aureus biofilms. [file mbio.00956-24-s0005.tif]
