# Supplementary material for: Pseudomonas aeruginosa surface motility and invasion into competing communities enhance interspecies antagonism
Source: mBio. 2024 Aug 6;15(9):e00956-24. doi: 10.1128/mbio.00956-24 (PMC11389416; doi:10.1128/mbio.00956-24)
Supplement: Supplemental Figures [file mbio.00956-24-s0006.pdf]

SUPPLEMENTARY FIGURES

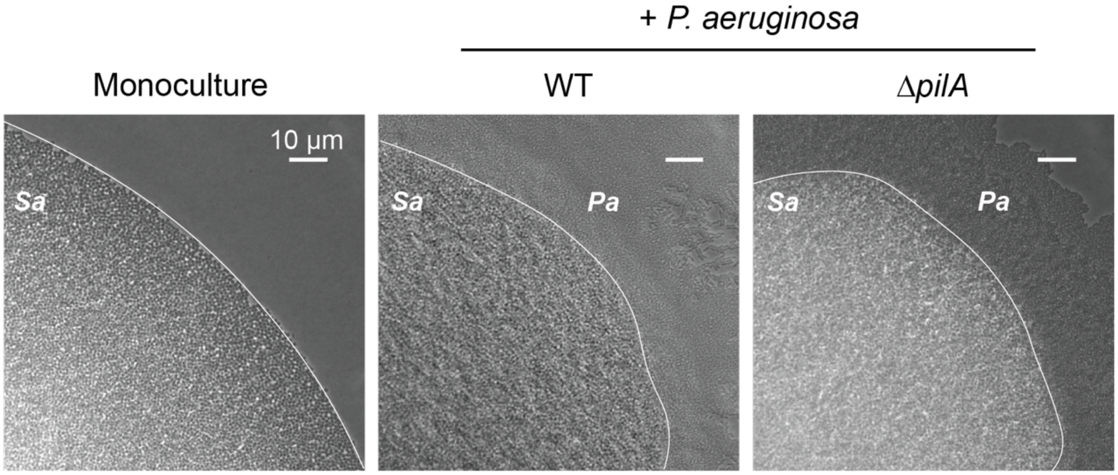

Figure S1. *P. aeruginosa* cells surround *S. aureus* colonies at the end time point. Representative phase contrast micrographs of *S. aureus* in mono- or coculture with WT or Δ*pilA P. aeruginosa* at ~24 hours. The white line traces *S. aureus* colony border.

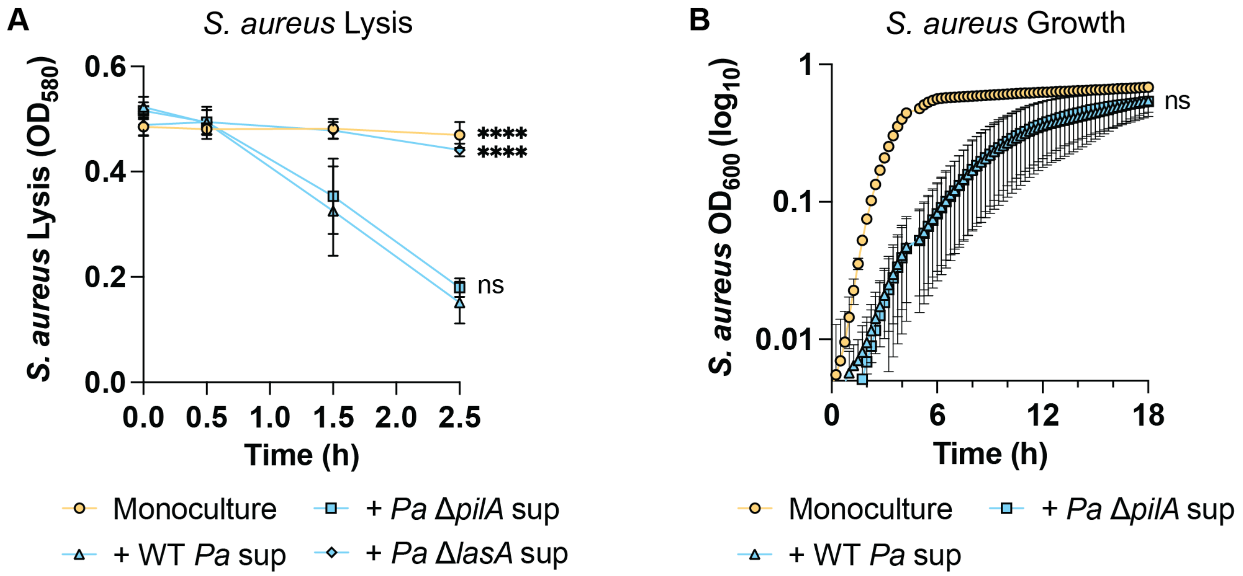

Figure S2. Exoproducts from Δ*pilA* lyse and inhibit *S. aureus* to the same levels as WT *P. aeruginosa* factors. *S. aureus* lysis (**A**) or growth (**B**) in the presence of WT, Δ*pilA*, or Δ*lasA P. aeruginosa* supernatant. Three (**A**) or four (**B**) biological replicates with three technical replicates each were performed. Statistical significance was determined by one-way ANOVA followed by Dunnett's Multiple Comparisons Test at 2.5 hours (**A**), or a two-way ANOVA followed by Tukey's Multiple Comparisons Test at every time point (**B**). No difference was found between the + WT *Pa* sup and the + *Pa* Δ*pilA* sup conditions in **B** at any time point. n.s., not significant; ****$P < 0.0001$.

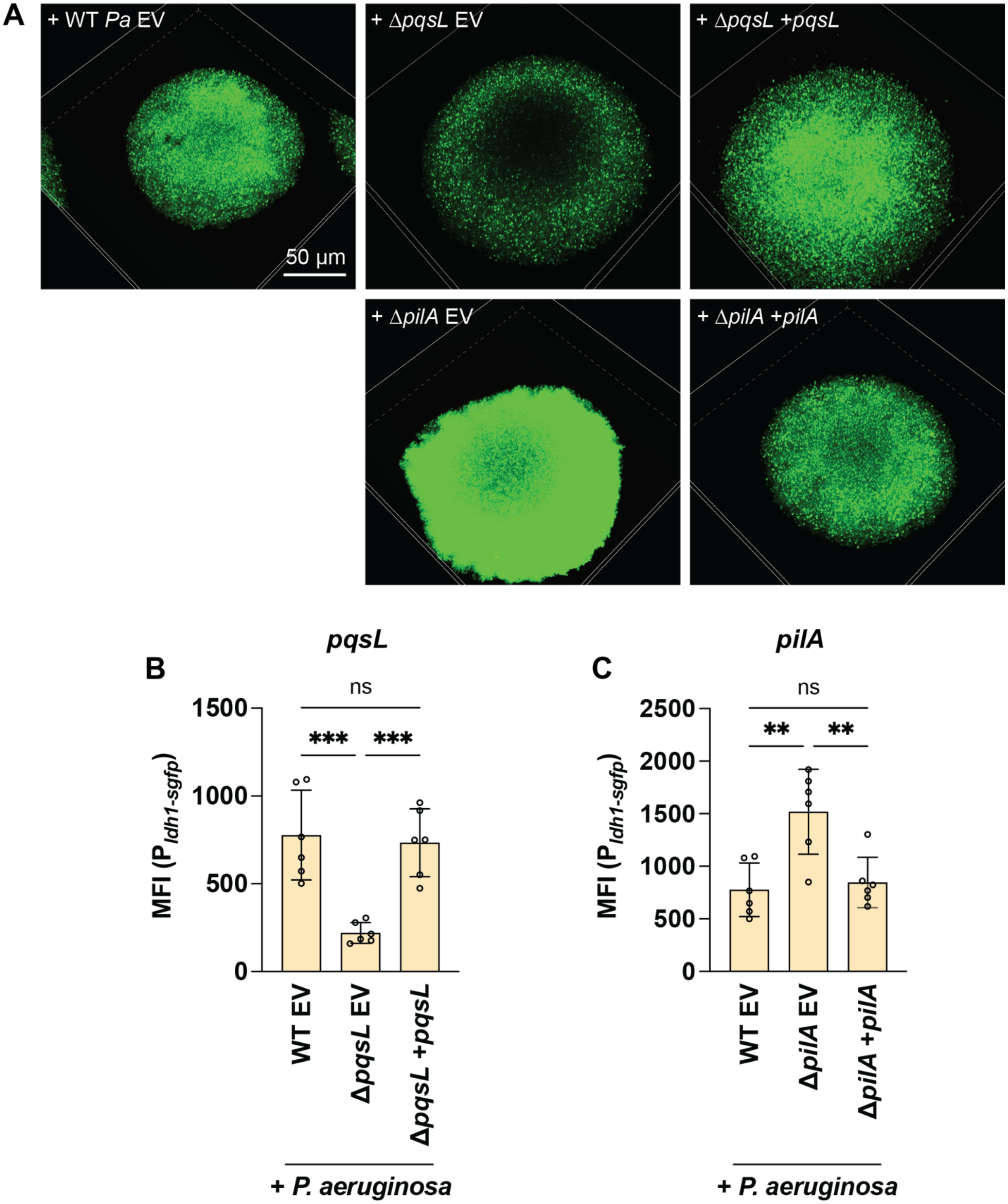

Figure S3. Genetic complementation of *pqsL* or *pilA*. *S. aureus* lactate fermentation ($P_{ldh1\text{-}sgfp}$) was measured in the presence of the indicated *P. aeruginosa* strains. (**A**) Representative resonant scanning confocal micrographs of *S. aureus* fermentation in coculture with WT *P. aeruginosa* pMQ72 P_{araBAD} empty vector (EV), Δ*pqsL* pMQ72 EV,

Δ*pqsL* pMQ72-$P_{araBAD\text{-}pqsL}$, Δ*pilA attTn7::*EV, or Δ*pilA attTn7::*$P_{araBAD\text{-}pilA}$ at *t* = 18 hours. (**B and C**) The mean fluorescence intensity (MFI) of *S. aureus* colonies was quantified in the presence of *P. aeruginosa pqsL* (**B**) or *pilA* (**C**) complementation strains at 18 hours for three biological replicates with two technical replicates each, and the mean and standard deviation are shown. Each data point represents one technical replicate. Statistical significance was determined by one-way ANOVA followed by Dunnett's Multiple Comparisons Test. n.s., not significant; $^{**}P < 0.01$ and $^{***}P < 0.001$.

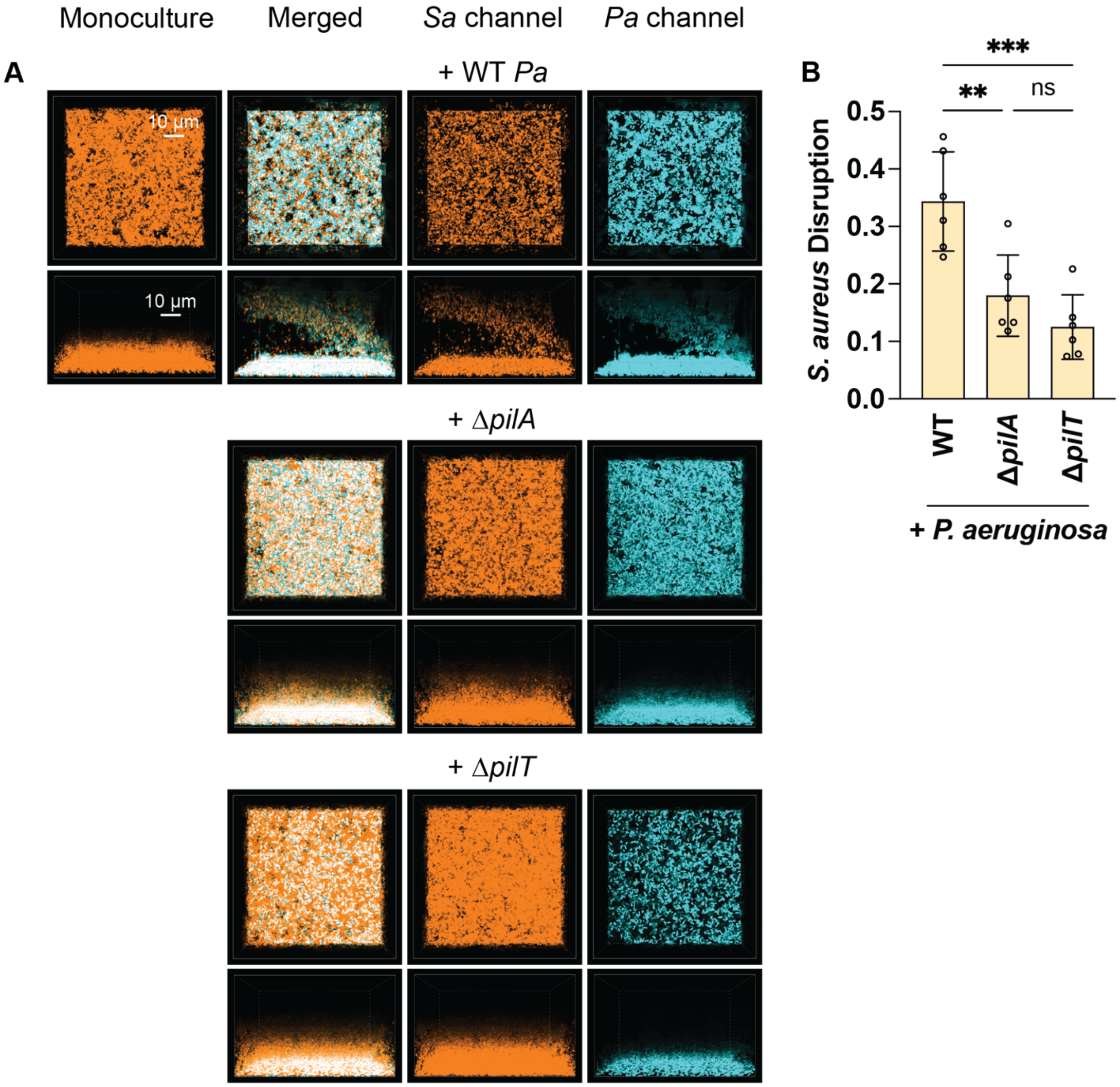

Figure S4. ***P. aeruginosa*** **type IV pili motility is necessary for disrupting pre-formed** ***S. aureus*** **biofilms.** (**A**) Representative resonant scanning confocal micrographs of *S. aureus* and *P. aeruginosa* in artificial sputum media. WT *S. aureus* (pseudocolored orange) in monoculture or in coculture with *P. aeruginosa* (pseudocolored cyan; WT, Δ*pilA*, or Δ*pilT*) t ~ 29 hours. (**B**) *S. aureus* biofilm disruption quantification (*Sa* volume at the coverslip / *Sa* volume at the top) at t ~ 29 hours. Three biological replicates with two technical replicates each were analyzed. The mean and standard deviation are shown. Each data point represents one technical replicate. Statistical significance was determined by one-way ANOVA followed by Dunnett's Multiple Comparisons Test. n.s., not significant; $^{**}P < 0.01$ and $^{***}P < 0.001$.
