# Supplementary material for: Pseudomonas aeruginosa surface motility and invasion into competing communities enhance interspecies antagonism
Source: mBio. 2024 Aug 6;15(9):e00956-24. doi: 10.1128/mbio.00956-24 (PMC11389416; doi:10.1128/mbio.00956-24)
Supplement: Supplemental Tables — Tables S1 to S3. [file mbio.00956-24-s0007.pdf]

SUPPLEMENTARY TABLES

Supplementary Table 1. Bacterial strains used in this study.

Strain	Alternative Strain Names	Genotype or Description	Reference or Source
Escherichia coli			
NEB® 5-alpha competent *E. coli*			New England Biolabs
S17 λpir	ECDHL23	*pro, res*$^-$ *hsdR17 (rK*$^-$ *mK*$^+$*) recA*$^-$ with an integrated *RP4-2-Tc::Mu-Km::Tn7*, Tp^r	(1)
Pseudomonas aeruginosa			
PA14	SMC232	Nonmucoid	(2)
PA14 Δ*pilA*	SMC3782		(3)
PA14 Δ*lasA*	PADHL447		This study
PA14 Δ*pqsL* Δ*pilA*	PADHL572		This study
PA14 mCherry	PADHL441	*attTn7::miniTn72.1-Gm-GW:P$_{A1/04/03\text{-}mCherry}$*; Gm^r.	This study
PA14 Δ*pilA* mCherry	PADHL439	*attTn7::miniTn72.1-Gm-GW:P$_{A1/04/03\text{-}mCherry}$*; Gm^r.	This study
PA14 Δ*pilT* mCherry	PADHL542	*attTn7::miniTn72.1-Gm-GW:P$_{A1/04/03\text{-}mCherry}$*; Gm^r.	This study
PA14 Δ*lasA* mCherry	PADHL461	*attTn7::miniTn72.1-Gm-GW:P$_{A1/04/03\text{-}mCherry}$*; Gm^r.	This study
PA14 Δ*pqsL* mCherry	PADHL442	*attTn7::miniTn72.1-Gm-GW:P$_{A1/04/03\text{-}mCherry}$*; Gm^r.	This study
PA14 Δ*pqsL* Δ*pvdA* Δ*pchE* mCherry	PADHL440	*attTn7::miniTn72.1-Gm-GW:P$_{A1/04/03\text{-}mCherry}$*; Gm^r.	This study
PA14 Δ*pqsL* Δ*pvdA* Δ*pchE* Δ*lasA* mCherry	PADHL463	*attTn7::miniTn72.1-Gm-GW:P$_{A1/04/03\text{-}mCherry}$*; Gm^r.	This study
PA14 Δ*pqsL* Δ*pvdA* Δ*pchE* Δ*lasA* Δ*pilA* mCherry	PADHL544	*attTn7::miniTn72.1-Gm-GW:P$_{A1/04/03\text{-}mCherry}$*; Gm^r.	This study

PA14 pMQ72-P$_{araBAD}$ empty vector	PADHL865	Gm^{r}	This study
PA14 Δ*pqsL* pMQ72-P$_{araBAD}$ empty vector	SMC6232	Gm^{r}	(4)
PA14 Δ*pqsL* pMQ72-P$_{araBAD-pqsL}$	SMC6231	Gm^{r}	(4)
PA14 Δ*pilA attTn7::*P$_{araBAD}$ empty vector	SMC7456	Gm^{r}	(5)
PA14 Δ*pilA attTn7::*P$_{araBAD-pilA}$	SMC7457	Gm^{r}	(5)
S. aureus			
USA300 JE2	SADHL05	USA300 CA-Methicillin resistant strain LAC without plasmids	(6)
JE2 pCM29	SADHL07	Cm^{r}	(7)
JE2 pEM87	SADHL167	Cm^{r}	(8)

Cm, chloramphenicol; Gm, gentamicin

Supplementary Table 2. Plasmids used in this study.

Plasmid	Description	Reference
P. aeruginosa		
pBT277 (pUC18-miniTn7T2-P$_{A1/04/03-mCherry}$)	Suicide vector with miniTn7 transposon with *mCherry* driven by the *A1/04/03* promoter; Ap^{r}, Gm^{r}.	(9)
pTNS3	Helper plasmid encoding the Tn7 transposase; Ap^{r}.	(10)
pEXG2-Tc-Δ*lasA*	Allelic exchange vector with *sacB* and *oriT* to delete *lasA* from PA14. Not temperature sensitive; Tc^{r}.	This study
pSMC259-Δ*pilA*	pMQ30 background. Allelic exchange vector with *sacB* and *oriT* to delete *pilA* from PA14; Gm^{r}.	(3)
pMQ72-P$_{araBAD}$ empty vector	Vector with arabinose-inducible gene expression system; Gm^{r}.	(11)
pMQ72-P$_{araBAD-pqsL}$	For arabinose-inducible expression of *pqsL*; Gm^{r}.	(4)
S. aureus		
pCM29	Plasmid with P$_{sarAP1-sgfp}$; Cm^{r}.	(7)
pEM87	Plasmid with P$_{ldh1-sgfp}$; Cm^{r}.	(8)

Ap, ampicillin; Cm, chloramphenicol; Gm, gentamicin; Tc, tetracycline

Supplementary Table 3. Primers used in this study.

Primer Name	Sequence (5'-3')	Description
ASP12_lasA_KO_HindIII_UP_F	GCATAAATGTAAAGCAAGCTTGCAGCCGCCGTCGGTT	This study

ASP13_lasA_KO_UP_R	ACTCAGAGCGCCAGGCGTGCTGCATGGGTAGCTCCTGGT	This study
ASP14_lasA_KO_DN_F	GCTACCCATGCAGCACGCCTGGCGCTCTGAGTCGGCGCG	This study
ASP15_lasA_KO_SacI_DN_R	TAAGGTACCGAATTCGAGCTCCTGGGCACGCCGATCGAATAC	This study
ASP18_lasA_KO_seq_Fwd	GCTGATAGTTGTCTTCCCGGGTGA	This study
ASP19_lasA_KO_seq_Rev	CCACTACGGCAAGGATGTGGTT	This study
oDHL34_pilA-check-F	CAAACCGAGAAGGTCGGACT	Lab collection
oDHL35_pilA-check-R	CACAAACAGATGATTGCCAGCA	Lab collection

SUPPLEMENTARY TABLES REFERENCES

1. Simon R, Priefer U, Puhler A. 1983. A broad host range mobilization system for *in vivo* genetic engineering: transposon mutagenesis in Gram negative bacteria. Bio-Technology 1:784-791.
2. Rahme LG, Stevens EJ, Wolfort SF, Shao J, Tompkins RG, Ausubel FM. 1995. Common virulence factors for bacterial pathogenicity in plants and animals. Science 268:1899-902.
3. Kuchma SL, Ballok AE, Merritt JH, Hammond JH, Lu W, Rabinowitz JD, O'Toole GA. 2010. Cyclic-di-GMP-mediated repression of swarming motility by *Pseudomonas aeruginosa*: The *pilY1* gene and its impact on surface-associated behaviors. J Bacteriol 192:2950-64.
4. Scott JE, Li K, Filkins LM, Zhu B, Kuchma SL, Schwartzman JD, O'Toole GA. 2019. *Pseudomonas aeruginosa* can inhibit growth of streptococcal species via siderophore production. J Bacteriol 201:e00014-19.
5. Kuchma SL, O'Toole GA. 2022. Surface-induced cAMP signaling requires multiple features of the *Pseudomonas aeruginosa* type IV pili. J Bacteriol 204:e0018622.
6. Miller LG, Perdreau-Remington F, Rieg G, Mehdi S, Perlroth J, Bayer AS, Tang AW, Phung TO, Spellberg B. 2005. Necrotizing fasciitis caused by community-associated methicillin-resistant *Staphylococcus aureus* in Los Angeles. N Engl J Med 352:1445-53.
7. Pang YY, Schwartz J, Thoendel M, Ackermann LW, Horswill AR, Nauseef WM. 2010. *agr*-dependent interactions of *Staphylococcus aureus* USA300 with human polymorphonuclear neutrophils. J Innate Immun 2:546-59.
8. Moormeier DE, Endres JL, Mann EE, Sadykov MR, Horswill AR, Rice KC, Fey PD, Bayles KW. 2013. Use of microfluidic technology to analyze gene expression during *Staphylococcus aureus* biofilm formation reveals distinct physiological niches. Appl Environ Microbiol 79:3413-24.
9. Zhao K, Tseng BS, Beckerman B, Jin F, Gibiansky ML, Harrison JJ, Luijten E, Parsek MR, Wong GCL. 2013. Psl trails guide exploration and microcolony formation in *Pseudomonas aeruginosa* biofilms. Nature 497:388-391.

10. Choi KH, Mima T, Casart Y, Rholl D, Kumar A, Beacham IR, Schweizer HP. 2008. Genetic tools for select-agent-compliant manipulation of *Burkholderia pseudomallei*. Appl Environ Microbiol 74:1064-75.
11. Shanks RM, Caiazza NC, Hinsa SM, Toutain CM, O'Toole GA. 2006. *Saccharomyces cerevisiae*-based molecular tool kit for manipulation of genes from Gram-negative bacteria. Appl Environ Microbiol 72:5027-36.
